# The role of sentrin-specific protease 2 substrate recognition in TGF-β-induced tumorigenesis

**DOI:** 10.1038/s41598-018-28103-8

**Published:** 2018-06-28

**Authors:** Che-Chang Chang, Yen-Sung Huang, Ying-Mei Lin, Chia-Ju Lin, Jen-Chong Jeng, Shin-Mei Liu, Tsai-Ling Ho, Ruei-Ting Chang, Chun A. Changou, Chun-Chen Ho, Hsiu-Ming Shih

**Affiliations:** 1 Graduate Institute of Translational Medicine, College of Medical Science and Technology, Taipei Medical University, Taipei, 11031 Taiwan; 2 The Ph.D. Program for Translational Medicine, College of Medical Science and Technology, Taipei Medical University, Taipei, 11031 Taiwan; 3 Ph.D Program in Biotechnology Research and Development, College of Pharmacy, Taipei Medical University, Taipei, 11031 Taiwan; 4 Traditional Herbal Medicine Research Center of Taipei Medical University Hospital, Taipei, 11031 Taiwan; 5 Institute of Biomedical Sciences, Academia Sinica, Taipei, 11529 Taiwan; 6 The Ph.D. Program for Cancer Biology and Drug Discovery, College of Medical Science and Technology, Taipei Medical University, Taipei, 11031 Taiwan; 7 Institute of Molecular and Genomic Medicine, National Health Research Institutes, Miaoli County, 35053 Taiwan

**Keywords:** Cancer, Cell biology

## Abstract

Smad4, a common-mediator of Smads, plays a central role in forming complexes with receptor-phosphorylated Smads, and then transduces transforming growth factor (TGF)-β signals into the nuclei. Although many cellular factors are involved in TGF-β induced epithelial-to-mesenchymal transition (EMT) and cell migration, very little is known with the mechanism of Smad4 regulation on pro-oncogenes response by TGF-β. Herein, we demonstrate the interaction of Sentrin-specific protease 2 (SENP2) with Smad4 through SENP2 residue 363~400. The same segment is also important for desumoylation of Smad4, and able to relieve sumoylation-mediated TGF-β repression. The SENP2^363~400^ segment is critical for TGF-β-induced cell migration, which is correlated with SENP2^363~400^ deletion mutant failed to increase matrix metalloproteinase (MMP)-9 and EMT marker gene expression. Moreover, our results suggest that the interaction and desumoylation between SENP2 and Smad4 promote cell migration in triple-negative breast cancer cells. Altogether, our data show how SENP2 regulates its substrate for desumoylation, and also the role of SENP2 in TGF-β induced cancer cell migration.

## Introduction

The transforming growth factor (TGF)-β family contains a large number of structurally related cytokines, including TGF-β, activins, inhibins, bone morphogenetic proteins (BMPs), and growth and differentiating factors (GDFs)^1–3^. TGF-β ligands are multitasking cytokines that regulate many different types of cellular processes, such as proliferation, growth inhibition, differentiation, cell migration, wound healing, and apoptosis^3^. These ligands act through heteromeric complexes of TGF-β type I and II serine/threonine kinase receptors and subsequently phosphorylate receptor-regulated (R)-Smads (such as Smad2 and Smad3). Activated R-Smads then form complexes with the common-mediator (Co)-Smad and are translocated into the nuclei to control target gene expression in TGF-β-induced cellular processes. Among Smad family members, Smad4, the only member of co-Smad, is a key intracellular mediator critical for transcriptional activation of TGF-β signaling. Therefore, regulation of the Smad4 expression level or activity is a critical issue for fine-tuning TGF-β signaling.

In the past decade, several studies indicated that overexpression or inactivation of TGF-β signaling components results in a bewildering array of effects associated with cancer suppression or progression. TGF-β functions as a tumor suppressor during early tumor outgrowth and switches towards promoting malignant conversion and progression at later stages^4^. In addition, TGF-β is a potent inducer of the epithelial- to-mesenchymal transition (EMT) in mammary cells, and this transformation is associated with acquisition of tumor stem-like properties, such as migration, metastasis, and mammosphere formation^5^. The TGF-β-induced EMT was enhanced with the overexpression of Smad2, Smad3, and Smad4 in NMuMg cells^6,7^, and mesenchymal-to-epithelial differentiation was induced by a TGF-β receptor kinase inhibitor^8^. TGF-β family members increase the formation of mammospheres in suspension culture, implying that TGF-β cytokines play a role in breast cancer progression^9^. Further studies indicated that TGF-β can have direct pro-oncogenic effects on tumor cells by stimulating their invasion and metastasis, and Smad4 is required for the TGF-β-induced EMT and bone metastasis of breast cancer cells^10,11^. Moreover, TGF-β could increase the cancer stem cell (CSC) population in a Smad4-dependent manner from clinical and preclinical data, and TGF-β signaling enhances tumor recurrence through interleukin (IL)−8-dependent expansion of CSCs in triple-negative breast cancer^12^. Altogether, Smad4 plays an important role in TGF-β-induced CSC formation and cancer progression in breast cancer.

Smad4 contains three functional domains: the MH1, Linker, and MH2 domains. Several lines of evidence indicate that post-translational modifications play pivotal roles in regulating the activity of Smad4. For example, Smad4 was constitutively phosphorylated in Mv1Lu and HSC4 cells^13^, which enhanced TGF-β-induced nuclear accumulation and the transcriptional activity of Smad4^14^. Moreover, ubiquitination of Smad4 by SCF^Skp2^ and SCF^β-TrCP1^ also interfered with TGF-β-dependent transcriptional activity and impaired the cell cycle arrest function^15,16^. Mono-ubiquitination of Smad4 occurs in the transcriptional activator complex and facilitates the turnover of Smad complexes at target genes, whereas poly-ubiquitination primarily occurs in unstable cancer mutants, leading to protein degradation^17,18^. In addition, Smad4 can also be conjugated by small ubiquitin-like modifier (SUMO)−1 at lysines 113 and 159, and mutation of K159 sumoylation residue significantly increases the Smad4 transcriptional potential via a reduction of interaction with a transcriptional corepressor Daxx, suggesting a negative regulatory mode of sumoylation on Smad4 activity^19^. Because the transactivation potential of Smad4 is regulated by sumoylation, to uncover the control of Smad4 sumoylation would be helpful in understanding cancer progression.

In this study, we demonstrated that Sentrin-specific protease 2 (SENP2), a SUMO-specific protease, interacts with Smad4 and deconjugates Smad4 sumoylation. Biochemical assay in both *in vitro* and *in vivo* indicated that SENP2^363~400^ section has bipartite interactions with Smad4 protein and SUMO molecule of sumoylated Smad4 protein, and it contributes to desumoylation of Smad4 protein and Smad response element-mediated transcriptional activation. In addition, SENP2 but not SENP2^363~400^ deletion mutant regulated TGF-β-induced cell migration, invasion and sphere formation. Taken together, our data suggest a notion that SENP2 regulates TGF-β/Smad4-dependent signaling and cellular functions via specific interactions and desumoylation of Smad4.

## Results

### SENP2 interacts with Smad4 and reduces Smad4 Sumoylation

**Figure 1:**
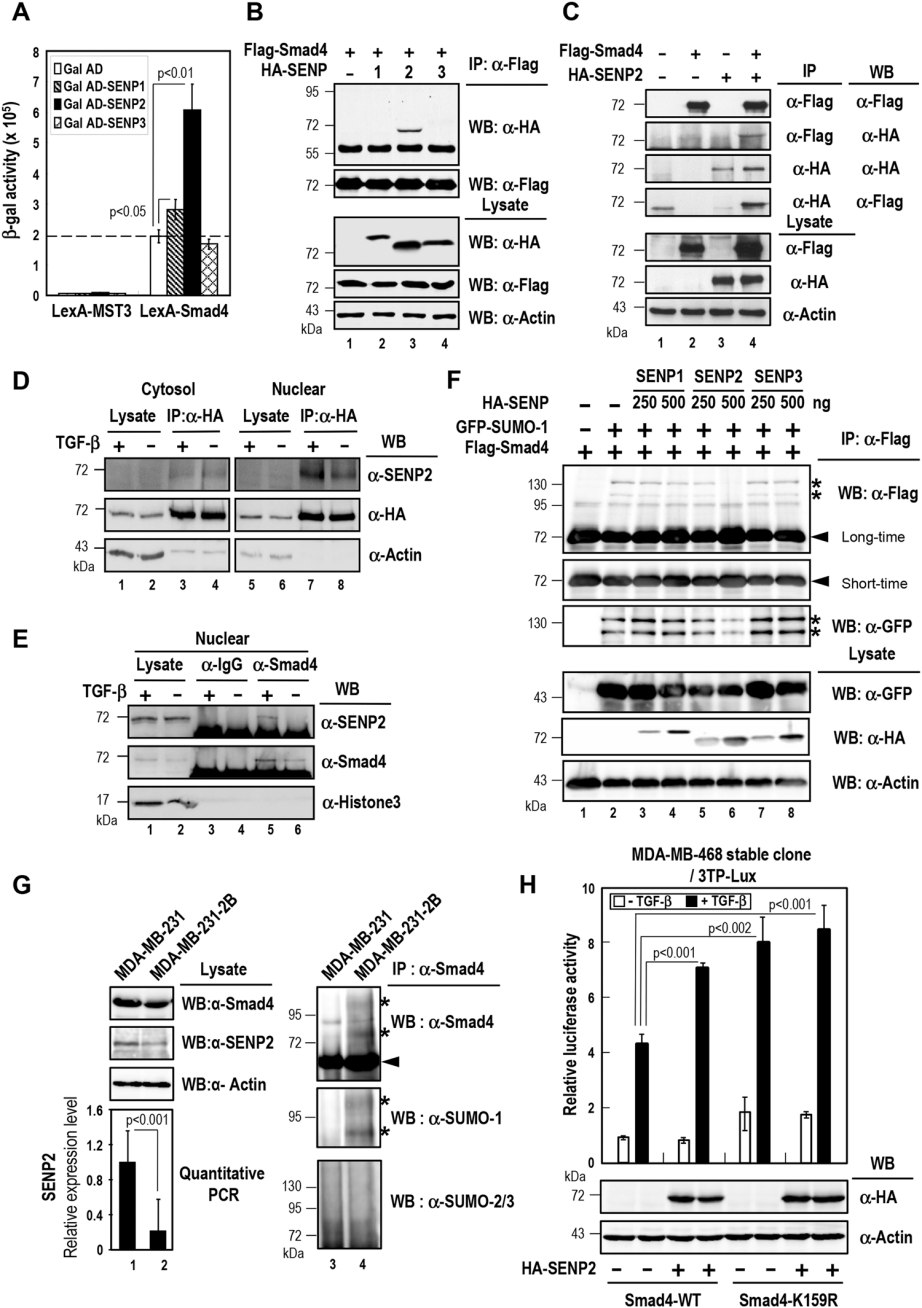
Interaction and desumolyation of Smad4 by SENP2, and effects on TGF-β-induced transcriptional potential. (**A**) *SENP2 interacts with Smad4 in yeast two-hybrid assays*. The L40 yeast strain was transformed with the plasmid constructs as indicated. The relative strength of protein interactions was determined by measuring β-galactosidase activities and normalized by cell density (A600). The data shown are means ± S.D. from three independent experiments performed in triplicate. Statistical significance was ascertained with Student’s *t*-test. (**B**,**C**) *SENP2 binds to Smad4 in vivo*. 293 T cells were transfected with indicated plasmid constructs for 48 h, and then harvested for analysis by immunoprecipitation (IP) and Western blot (WB) using anti-HA and anti-Flag antibodies. For measuring the expression levels of transfected proteins, 20 μg of cell lysates was subjected to immunoblotting with indicated antibody (bottom three panels). (**D**) *Smad4 interacts with SENP2 in cell nucleus*. The cytosol and nuclear fraction of HA-Smad4 overexpressed MDA-MB-231 cells were lysed, immunoprecipitated with anti-HA antibody and followed by WB analysis with anti-SENP2 and anti-HA antibodies. The expression level of HA-tagged Smad4 and SENP2 was measured by WB analysis. (**E**) *TGF-β treatment facilities the endogenous Smad4/SENP2 interaction*. The nuclear fraction of MDA-MB-231 cells was lysed, immunoprecipitated with anti-Smad4 antibody and followed by WB analysis with anti-SENP2 antibody. The expression level of Smad4 and SENP2 was measured by immunoblotting. (**F**) *Smad4 is de-sumoylated by SENP2*. 293 T cells were transfected with expression vector for 1.5 μg of Flag-Smad4 and 0.5 μg of GFP-SUMO-1, together with increasing amounts of expression plasmids for HA-SENP1, SENP2 or SENP3 as indicated. The transfected cells were lysed and subjected to immunoprecipitation with anti-Flag antibody. Western blots analysis with anti-Flag and anti-GFP antibodies show sumoylation levels of Flag-tagged Smad4. The expression levels of GFP-SUMO-1 and HA-SENPs were determined by WB analysis (bottom three panels). *Asterisk* and *arrowhead*, SUMO-1-modified and -unmodified Smad4 proteins, respectively. (**G**) SENP2 knockdown increases the SUMO-1 conjugated Smad4. The lysates of MDA-MB-231 or MDA-MB-231-2B cells were immunoprecipitated with anti-Smad4 antibody and followed by WB analysis with anti-Smad4, anti-SUMO-1 and anti-SUMO2/3 antibodies. Western blotting and an RT-qPCR were used to analyze SENP2 expression level. Statistical significance was ascertained with Student’s *t*-test. *Asterisk* and *arrowhead*, SUMO-1-modified and -unmodified Smad4 proteins, respectively. (**H**) *Promotion of TGF-β-induced reporter activity by SENP2*. MDA-MB-468 Smad4 stable cells were transfected with 3TP-Lux and TK-Renilla reporter construct with HA-SENP2 plasmids as indicated. After transfection, the cells were starved for 24 h followed by TGF-β treatment for 24 h. The cells were harvested and subjected to reporter assays as described in “Material and Method”. Relative luciferase activity is represented as the means ± S.D. from three independent experiments. Statistical significance was ascertained with Student’s *t*-test.

We previously showed that Smad4 can be SUMO-modified, leading to the recruitment of Daxx in repressing TGF-β/Smad4-induced signaling^20^. These findings suggest that sumoylation and desumoylation of Smad4 are important for fine-tuning TGF-β/Smad4-mediated cellular events. To identify SENP family proteins involved in desumoylation of Smad4, we performed yeast two-hybrid assays using LexA-Smad4 as bait with Gal-AD fused to SENP1, SENP2, or SENP3 as prey. In Fig. 1A, LexA-Smad4 gave a robust interaction with Gal-AD-SENP2, while it showed modest and no interaction with Gal-AD-SENP1 and Gal-AD-SENP3, respectively. These results suggest that SENP2 specifically interacts with Smad4. Moreover, both co-immunoprecipitation (IP) experiment (Figs 1B and S1A), and reciprocal IP experiment (Fig. 1C) showed similar interaction between Smad4 and SENP2, further confirmed that SENP2 can form protein complexes with Smad4 in cells.

We next determined the location of Smad4/SENP2 complexes in cell. It has been reported that SENP2 is associated with the nuclear pore and located on nucleoplasmic side of the nuclear pore^21^. Indeed, the results of subcellular fraction and immunostaining showed that SENP2 was localized in the nuclear compartment and partly interacted with Smad4 (Fig. 1D, lane 8 and S1B). With TGF-β treatment, Smad4 was translocated into the nuclear region to form complexes with SENP2 (Fig. 1D, lane 7 and S1B). In fact, the interaction of endogenous Smad4 and SENP2 was significantly increased with TGF-β treatment (Fig. 1E, lane 5 vs lane 6), and similar results were observed in the nuclear fraction of MDA-MB-231.

We further examined whether SENP2 could desumoylate Smad4. Plasmids expressing epitope-tagged Smad4 and GFP-SUMO-1 were cotransfected with increasing level of epitope-tagged SENP1, SENP2, SENP3 or SENP5 into HeLa cells. Co-expression of GFP-SUMO-1 yielded two sumoylated Smad4 bands (Fig. 1F, lane 2 vs lane 1 indicated by *asterisk*). Notably, both bands were decreased by cotransfection of the SENP2, but not with SENP1, SENP3 or SENP5 (Fig. 1F, lanes 3–8 and Fig. S1C, lanes 3–4). To further confirm the potential desumoylation function of SENP2 on Smad4, we created SENP2 knockdown MDA-MB-231 breast cancer cell line (MDA-MB-231-2B) by CRISPR-Cas9 approach (Fig. 1G, lanes 1-2 and S1D). As shown in Fig. 1G, the SUMO-1 conjugated Smad4 was increased in SENP2 knockdown cell, but not SUMO-2/3 conjugated Smad4. In line with these findings, SENP2 but not SENP1 or SENP3 enhanced the TGF-β-induced transcriptional potential in a reporter activity assay (Fig. S1E). More importantly, SENP2-mediated increment of the TGF-β-induced reporter activity was abolished in Smad4 K159R stable cells (Fig. 1H). Altogether, these results suggested that SENP2 upregulates TGF-β-induced reporter activity through Smad4 desumoylation.

### SENP2^363~400^ mediates substrate interaction

**Figure 2:**
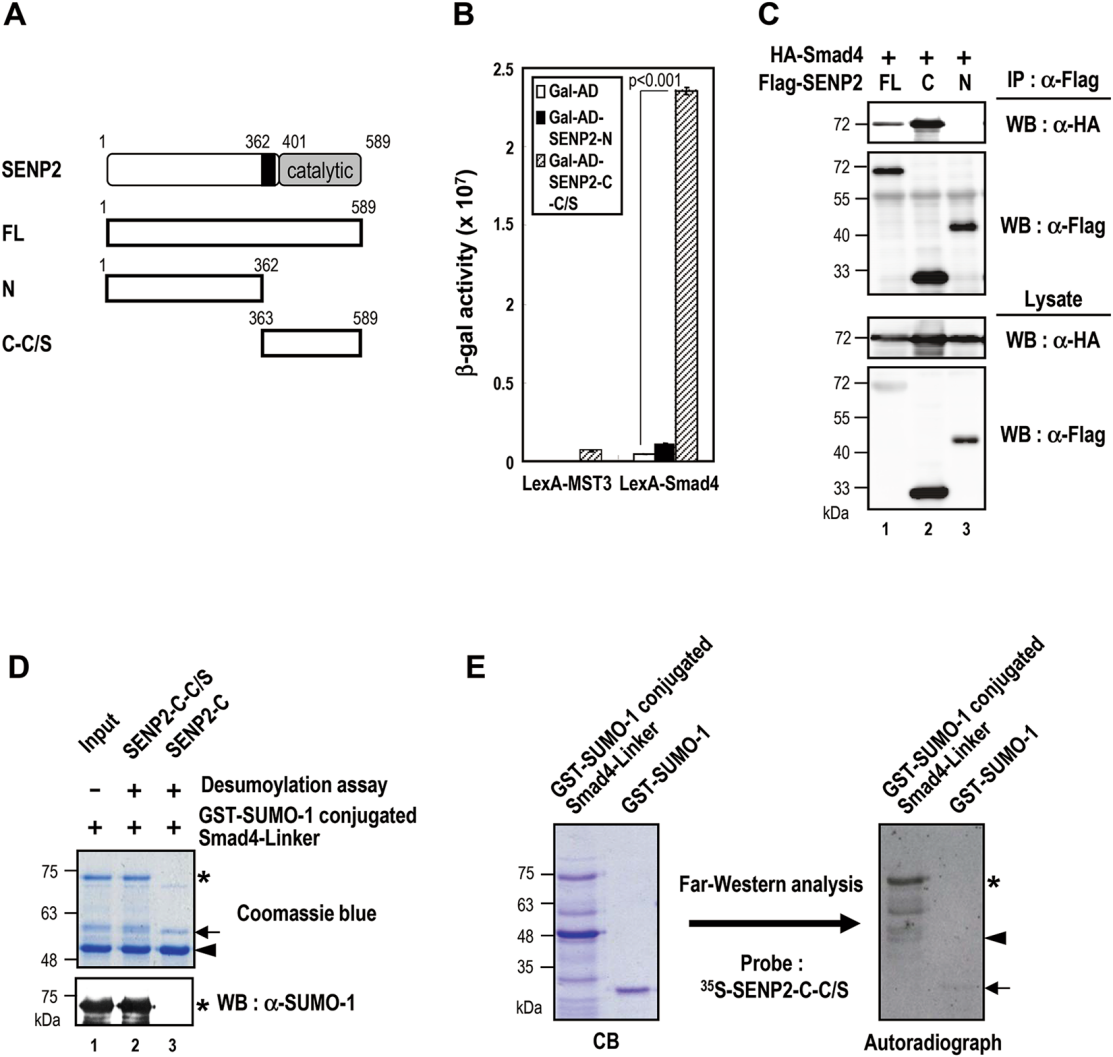
SENP2^363~589^ participates in the Smad4 interaction and desumoylation. (**A**) *Schematic representation of SENP2 deletion mutants used in this study*. FL, full-length SENP2; N, aa 1~362 of SENP2; C-C/S, aa 363~589 of SENP2. (**B**) *SENP2*^*363-589*^ *interacts with Smad4 in yeast two-hybrid assays*. Quantitative β-gal analyses of L40 yeast cells co-transformed with the indicated constructs. The data shown are the means ± S.D. from three independent experiments performed in triplicate. Statistical significance was ascertained with Student’s *t*-test. (**C**) *SENP2*^*363-589*^ *interacts with Smad4 in vivo*. 293 T cells were transfected with indicated plasmid constructs for 48 h, and then harvested for analysis by immunoprecipitation (IP) and Western blot (WB) using anti-HA and anti-Flag antibodies. Western blotting was used to analyze HA-Smad4 and Flag-SENP2 variants expression level. (**D**) *SENP2*^*363~589*^ *is sufficient for Smad4 desumoylation in vitro*. An *in vitro* desumoylation assay was performed for 30 min at 30 °C with the recombinant GST-sumoylated Smad4-Linker protein and GST-SENP2-C or GST-SENP2-C-C/S. Reaction products were analyzed by Coomassie blue staining and immunoblotting with an anti-SUMO-1 antibody. *Asterisk* and *arrowhead*, SUMO-1-modified and -unmodified GST-Smad4 Linker protein, respectively. *Arrow*, GST-SENP2-C or -C-C/S protein. (**E**) *SENP2*^*363~589*^ *binds Smad4-Linker and SUMO-1 modified Smad4-Linker protein both*. Autoradiographs showed that ^35^S-labeled SENP2-C-C/S bind to recombinant GST-Smad4-Linker, GST-sumoylated Smad4-Linker, and the GST-SUMO-1 protein. *Asterisk* and *arrowhead*, SUMO-1-modified and -unmodified GST-Smad4 Linker protein, respectively. *Arrow*, GST-SUMO-1 protein.

Since SENP2 can interact and desumoylate Smad4, we next delineated the subdomain(s) of SENP2 involved in Smad4 recognition. Both SENP2 regulatory domain (aa 1~362) and catalytic domain with active site CS mutation (Gal-AD-SENP2-C-C/S aa 363~589) were subjected to yeast-two-hybrid assay. We used the catalytic activity defective mutant to avoid toxicity of SENP2 catalytic domain for yeast growth. Smad4 interacted with SENP2-C-C/S, but not SENP2-N, in yeast (Fig. 2B). Likewise, SENP2-C, but not SENP2-N, formed complexes with Smad4 in a co-IP experiments (Fig. 2C), indicating the C-terminal domain of SENP2 responsible for Smad4 interaction.

Because SENP2 recognizes sumoylated substrates for desumoylation, we next examined whether SENP2-C is sufficient to bind sumoylated Smad4. We first generated K159 sumoylated Smad4 recombinant protein from *in vitro* sumoylation reaction of Smad4 linker domain, which can be identified as a band slowly migrating below molecular weight maker 75 kDa (Fig. 2D, lane 1, *asterisk*). As expected, this band could be removed by adding SENP2-C WT but not CS mutant (Fig. 2D, lanes 2 and 3). The sample containing sumoylated Smad4 was subjected to Far-Western analysis using *in vitro* synthesized ^35^S-labled SENP2-C-C/S as a probe. Notably, SENP2 C-terminal domain bound to the sumoylated Smad4 (*asterisk*) more robust than the un-sumoylated Smad4 (*arrowhead*) or SUMO-1 (*arrow*) (Fig. 2E, right panel). The amount of each protein in the input was shown by commassie blue staining (left panel). That fact that SENP2-C preferentially binds to sumoylated Smad4 protein, strongly suggests that SENP2-C consists of bipartite interactions with sumoylated Smad4.

**Figure 3:**
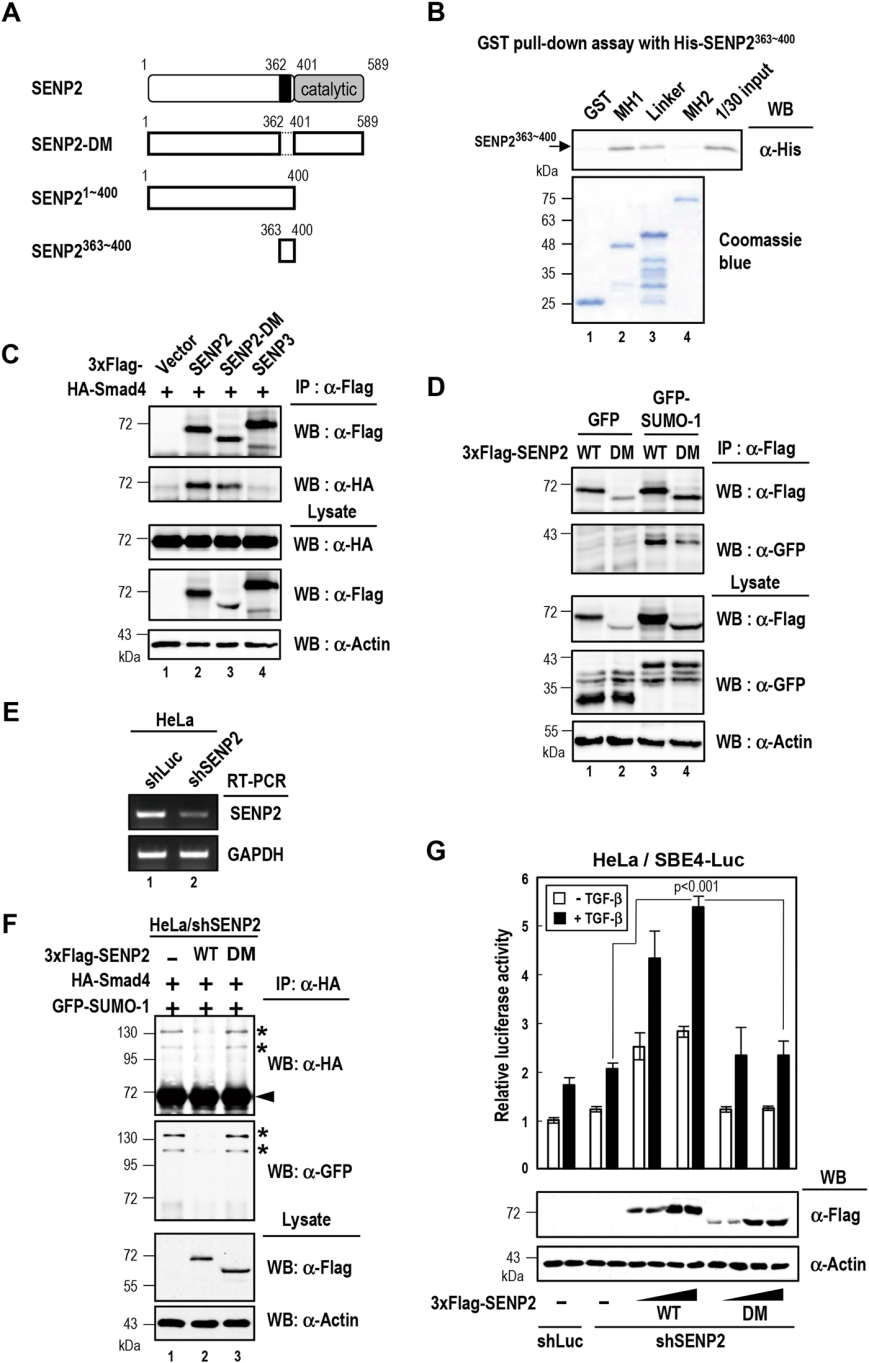
SENP2^363~400^ mediates Smad4 binding. (**A**) *Schematic representation of SENP2 deletion mutants used in this study*. SENP2-DM, SENP2^363~400^ deletion mutant. SENP2^1~400^, aa 1~400 of SENP2; SENP2^363~400^, aa 363~400 of SENP2. (**B**) *SENP2*^*363-400*^ *binds Smad4-MH1 and Linker region in vitro*. The recombinant His-SENP2^363~400^ was pulled down by GST or GST-Smad4 subdomain protein. The reaction products were analyzed by immunoblotting with anti-His antibody. (**C**) *SENP2-DM reduced the Smad4 interaction*. HeLa cells were transfected with indicated plasmid constructs for 48 h, and then harvested for analysis by immunoprecipitation (IP) and Western blot (WB) using anti-HA and anti-Flag antibodies. The expression levels of transfected proteins were measured by immunoblotting with indicated antibody. SENP3 was as negative control for IP. (**D**) *SENP2-DM had SUMO-1 binding ability*. IP and WB were used to analyze the SUMO-1 binding activity of SENP2 and SENP2-DM. The expression levels of transfected proteins were measured by immunoblotting with indicated antibody. GFP was as negative control for IP. (**E**) *Knockdown of SENP2 with lentivirus carrying SENP2 shRNA.* Semiquantitative RT-PCR was used to analyze SENP2 expression in pLKO-shLuc- or pLKO-shSENP2-treated HeLa cells. GAPDH was used as the expression control. (**F**) *The SENP2*^*363~400*^ *is crucial for desumoylation of Smad4*. The SENP2 knockdown cells were transfected with indicated plasmid. The resulted cell lysates were IPed with anti-HA antibody followed by Western blot analysis with indicated antibodies. Western blots analysis with anti-HA and anti-GFP antibodies show sumoylation levels of HA-tagged Smad4. The expression levels of Flag-SENP2 and SENP2-DM were determined by WB analysis. *Asterisk* and *arrowhead*, SUMO-1-modified and -unmodified Smad4 proteins, respectively. (**H**) *SENP2-DM incapables to active TGF-β-induced transcriptional potential*. HeLa shSENP2 stable cells were transfected with the SBE4-Luc and TK-Renilla reporter construct with 3xFlag-SENP2 or SENP2-DM plasmids as indicated. After transfection, the cells were starved for 24 h followed by TGF-β treatment for 24 h. The cells were harvested and subjected to reporter assays as described in “Material and Method”. Relative luciferase activity is represented as the means ± S.D. from three independent experiments. Statistical significance was ascertained with Student’s *t*-test.

Since catalytic domain of SENP2 (containing residues 401~589) is able to bind SUMO protein^22^, we then tested whether SENP2 protein segment 363~400 was involved in Smad4 interaction. The results of co-IP experiments showed that SENP2 residues 1~400 formed complexes with Smad4 (Fig. S2A), suggesting that segment 363~400 is essential and sufficient to associate with Smad4. In additional, this segment could bind to Smad4 linker and MH1, but not MH2 domain, by GST pull down assays (Fig. 3B), correlating with both sumoylation sites situated within MH1 domain and linker region. As expected, SENP2 deletion mutant of 363~400 segment (SENP2-DM) showed reduced Smad4 interaction (Fig. 3C, lanes 2 and 3). Noted that such deletion mutant retained the capacity in SUMO-1 interaction (Fig. 3D). These results further support the notion that SENP2 recognizes sumoylated Smad4 substrate via bipartite interactions.

We next examined whether SENP2^363~400^ is important for Smad4 desumoylation by *in vivo* desumoylation assay. We first depleted endogenous SENP2 protein in HeLa cells (Fig. 3E), then subsequently transfected SENP2-DM into SENP2-depleted cells. Notably, the level of Smad4 sumoylation remained the same with SENP2-DM, but reduced with WT SENP2 (Fig. 3F). In addition, these results indicate that SENP2^363~400^ is important for Smad4 recognition and it is essential for sumoylated-Smad4 deconjugation. In line with this notion, SENP2-DM could not potentiate the TGF-β-induced report gene activity (Figs 3G and S2B).

### SENP2 modulates cell migration and sphere formation via Smad4 interaction and desumoylation

**Figure 4:**
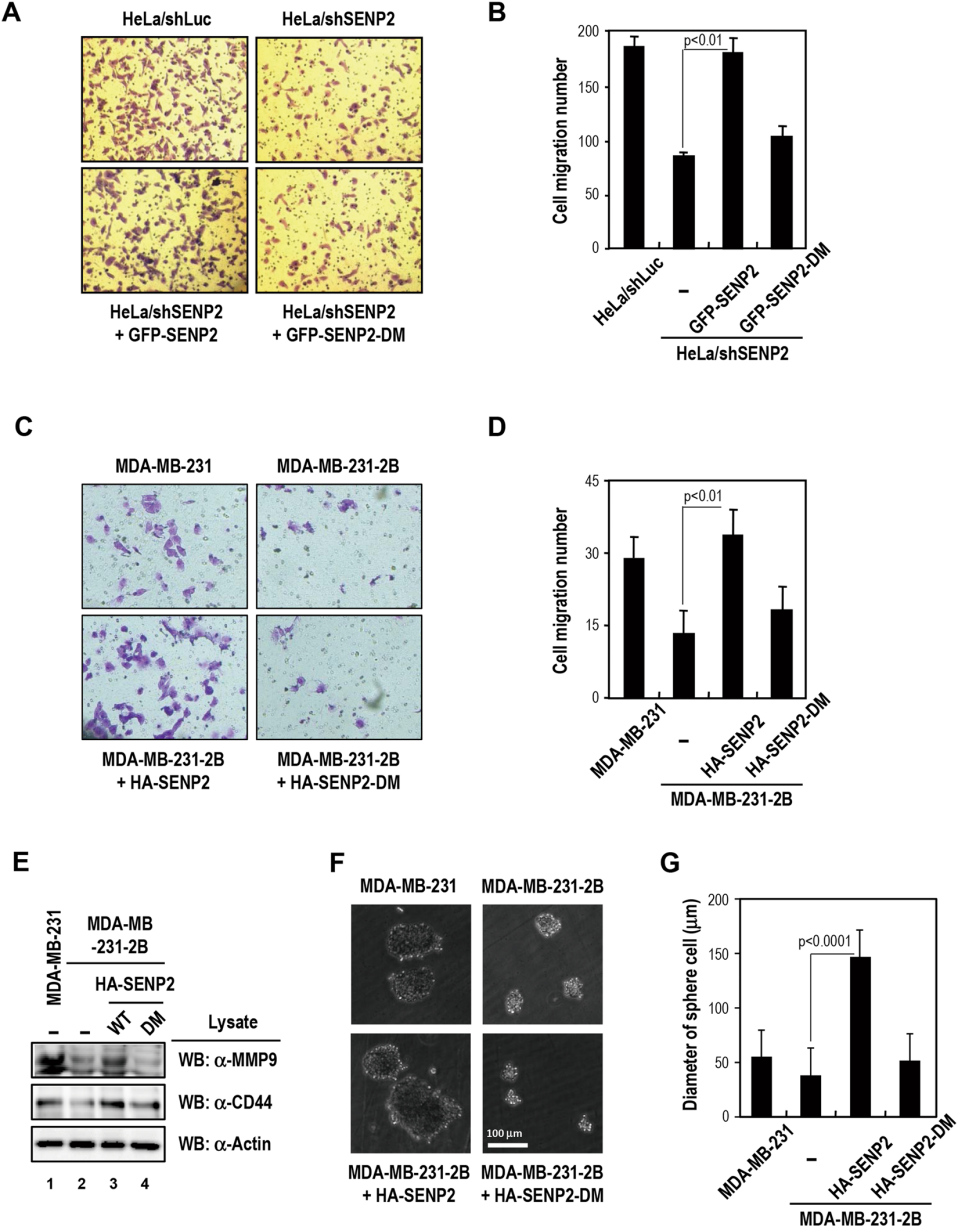
SENP2 is involved in cell migration and sphere formation. (**A**,**B**) *SENP2 promotes the migration of HeLa cell*. SENP2-knockdown HeLa cells were transfected with the indicated plasmid and subjected to measure the cell migratory ability using a Boyden chamber analysis with 10% FBS in the low compartment. Migrating cells were analyzed by a crystal violet assay (A) and counted with microscopy (B). Error bars show standard deviations from 10 microscopic fields from three independent experiments. Statistical significance was ascertained with Student’s *t*-test. (**C**,**D**) *SENP2 promotes the migration of MDA-MB-231 cell*. MDA-MB-231-2B cells were transfected with the indicated plasmid and subjected to measure the cell migratory ability using a Boyden chamber analysis. The migratory ability was determined as above. (**E**) *The SENP2 enhances the expression of MMP9 and CD44*. MDA-MB-231-2B cells were re-introduced HA-SENP2 or SENP2-DM plasmid. The resulted cell lysates were used to analyze the expression level of MMP9 and CD44 by immunoblotting with indicated antibodies. (**F**,**G**) *SENP2 involves in sphere cell formation*. MDA-MB-231-2B cells were transfected with the indicated plasmid and used MEGM medium to culture sphere cell (F). The diameters of sphere cell were measured with ImageJ software (G). Error bars show standard deviations from ≥100 sphere cells in three experiments. Statistical significance was ascertained with Student’s *t*-test.

We next explored the role of SENP2 in TGF-β signaling-regulated cellular processes, such as cell migration and sphere formation. We performed a transwell migration assay with cells expressing SENP2 or SENP2-DM. SENP2-knockdown HeLa cells exhibited ~50% decrease in mobility, and this could be rescued by exogenous SENP2 but not SENP2-DM (Figs 4A, B and S2A). Similarly, MDA-MB-231-2B cells also showed reduction in cell migration, and re-introduction of SENP2, but not SENP2-DM, fully restored the capacity (Figs 4C, D and S3C). More importantly, SENP2 and SENP2-DM re-introduced cells exhibited similar cell proliferation rate, indicating the reduction of cell migration was not due to the difference in cell viability (Figs S3B and S3D). These results suggest that SENP2 regulates cell migration via Smad4-interacting segment. In addition, previous report has showed that Smad4 activates MMP9 expression related to EMT pathway^23^. We then examined whether MMP9 expression is also regulated by SENP2. Notably, MDA-MB231-2B cell showed significantly lower MMP9 expression (Fig. 4E, lanes 1 and 2), and re-introduction of SENP2, but not SENP2-DM, restored MMP9 levels (lanes 3 and 4). Similar observation was made with SENP2-depleted HeLa cells (Fig. S3A). In addition, we also found that SENP2, but not SENP2-DM, could increase sphere formation, correlating with the expression level of cluster of differentiation 44 (CD44) (Fig. 4E–G). These data provided strong correlations between SENP2 in regulating Smad4 target gene expression, cellular migration, and sphere formation.

### SENP2 modulates TGF-β-induced cell migration and invasion via SENP2^363~400^ segment

**Figure 5:**
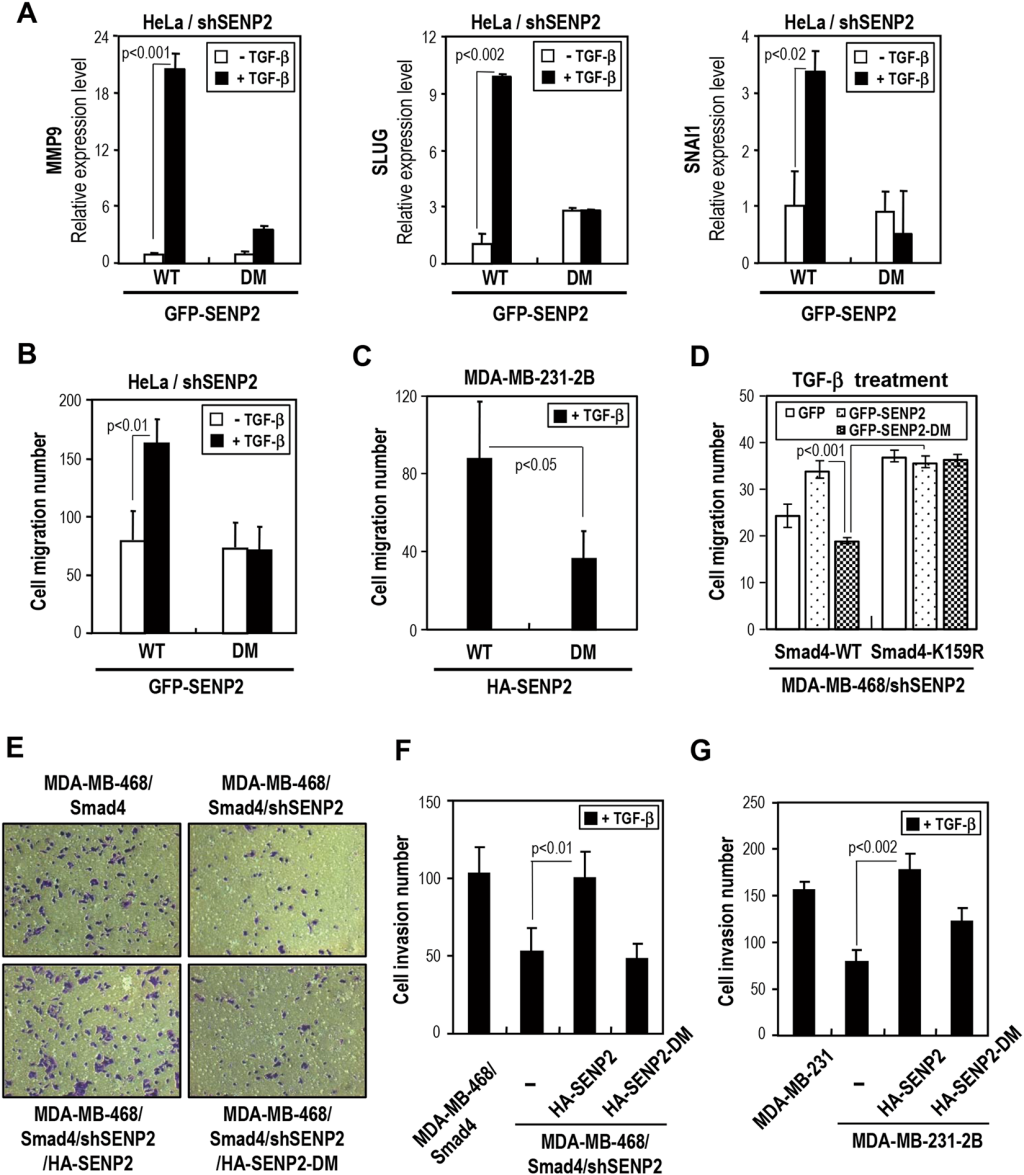
SENP2 modulates TGF-β-induced cell migration via the Snail/Slug/MMP9 axis. (**A**) *Quantitative RT-PCR of gene expression levels in the Snail/Slug/MMP9 axis*. GFP-SENP2 and GFP-SENP2-DM-reintroduced shSENP2 HeLa cells were starved for 24 h and with/without TGF-β treatment for 24 h. RT-qPCR were used to analyze Snail, Slug and MMP9 expression. Relative expression levels of individual genes were calculated and results are shown as folds of change. Results are presented as the mean ± SD, *n* = 3. (**B,C**) *SENP2 increases the TGF-β-induced cell migration*. The SENP2 knockdown HeLa cells (B) and MDA-MB-231-2B cells (C) were re-introduced with indicated plasmid, subjected to Boyden Chamber and starved for 24 h. With TGF-β treatment in the low compartment for 24 h, the cell migratory ability was determined by microscopy with crystal violet stain. Error bars represent standard deviations from three independent experiments. Statistical significance was ascertained with a Student’s *t*-test. (**D**) *Desumoylation of Smad4 contributes to TGF-β induced cell migration*. Cell migration assay with MDA-MB-468 Smad4 variant stable cells as indicated. The cells were starved for 24 h followed by TGF-β treatment for 24 h and the cell migratory ability was determined as described above. (**E**–**G**) *SENP2 involves in TGF-β induced cell invasion*. MDA-MB-468/Smad4/shSENP2 and MDA-MB-231-2B cells were re-introduced with HA-SENP2 or SENP2-DM plasmid, subjected to Matrigel-coated Boyden Chamber and starved for 24 h. With TGF-β treatment in the low compartment for 24 h, the invasion cells were analyzed by a crystal violet assay (E) and counted with microscopy (F,G). Error bars show standard deviations from 10 microscopic field in three experiments. Statistical significance was ascertained with Student’s *t*-test.

It has been reported that Smad4 is required for TGF-β-induced EMT and bone metastasis of breast cancer cells^10^. Besides, MMPs play important roles in TGF-β-induced migration and invasion^24^. We further tested whether Smad4-medated target genes associated with EMT pathway are also regulated by SENP2 under TGF-β treatment. The results of RT-qPCR quantification analyses showed that SENP2 but not SENP2-DM could mediate TGF-β-induced expression of the MMP9, Snail, and Slug genes (Fig. 5A). Interestingly enough, similar pattern could also be found with cell migration phenotype in HeLa cells (Fig. 5B) and MDA-MB-231-2B cells (Figs 5C and S4). All together, our findings indicated that SENP2 is involved in TGF-β-induced cell migration through EMT gene expression, which is achieved by reducing Smad4 sumoylation-mediated gene repression.

To further substantiate this notion of SENP2 in regulating cell migration via desumoylation of Smad4, we introduced Smad4 WT or K159R mutant into MDA-MB-468 cell line, which lacks endogenous Smad4 expression. The resulting cells were further subjected to SENP2 depletion by shRNAs, and followed by rescue experiments with SENP2 or SENP2-DM under TGF-β treatment. As expected, SENP2 re-expression could promote cell migration in Smad4 WT cells but not in Smad4 K159R cells (Fig. 5D).

We next examined the SENP2^363~400^ segment function in cell invasion. The Smad4 introduced MDA-MB-468 cells were depleted by SENP2 shRNA and subjected to transfection with SENP2 or SENP2-DM. With the TGF-β treatment, the SENP2 re-introduced cells have higher invasion ability than SENP2-DM re-introduced cells (Figs 5E and F). In addition, similar results were observed in MDA-MB231-2B cells (Fig. 5G). Altogether, our data strongly suggest that SENP2 regulates TGF-β-induced cell migration and invasion via Smad4 interaction and desumoylation.

## Discussion

SUMO binding and substrate recognition are key steps in the process of protein desumoylation. Previously, structure-based studies of SENP2 with sumoylated RanGAP1 revealed that SENP2 catalytic domain contributes to distinct surfaces for sumoylated protein interactions. One surface is primarily for globular SUMO domain interaction, and the other is to form the substrate exit tunnel to provide additional contact for substrate lysine and SUMO consensus site^22,25^. In this study, we demonstrated that SENP2 binds to sumoylated Smad4 substrate behind the catalytic domain. We identified SENP2 segment 363~400, which is important to mediate Smad4 substrate interaction. Deletion of SENP2 363~400 still retained the capacity of the SENP2 catalytic domain for SUMO interaction (Figs 3B and C), consistent with the findings from structural studies. In addition, SENP2 363~400 could bind to Smad4 protein domains MH1 and linker region, but not MH2 domain, further suggesting that the segment 363~400 is important for substrate interaction. In additional to the SENP2 catalytic domain in SUMO and SUMO-conjugated lysine interactions, we propose that SENP2 363~589 consists of bipartite interactions with sumoylated substrates. Noted that SENP2 363~400 amino acid residues are different from those of other SENP family proteins such as SENP1. Whether SENP2 substrate specificity is through the 363~400 segment remains further investigation.

Recent studies indicated that dysregulation of sumoylation is involved in cancer cell proliferation, migration, and invasion^26,27^. Sumoylation components and levels are increased in cells under stress^28,29^. In addition, similar results were observed in tumor samples, especially sumoylation of the E2-ligating enzyme, Ubc9. However, the Ubc9 expression level was higher in primary tumors but lower in metastatic tumors^30^. Coincidentally, several studies reported that the expression of SENP family proteins was increased in cancer tissues^31–34^. These findings suggest that the dynamic reaction between sumoylation and desumoylation is regulated during cancer progression. Our findings indicated that SENP2 plays a positive role in Smad4 desumoylation to promote breast cancer cell migration and sphere formation. Interestingly, Kaplan-Meier plots showed that a high level of SENP2 gene expression was significantly associated with poor survival in basal-like breast cancer patients (*p* = 0.026) (Fig. S5). These results may provide a correlation of SENP2 with breast cancer progression.

On the other hand, recent studies of SENP2 in bladder cancer showed that the expression of SENP2 is downregulated in bladder cancer cells, causing an increase of MMP13 for cell migration and invasion^35,36^. In this scenario, SENP2 desumoylates TBL1/TBLR1, resulting in an inhibition of β-catenin translocation into the nucleus for MMP13 gene activation. The distinct outcomes of SENP2 in regulating cell migration and invasion could be due to different cellular contexts of cancer cells. Consistent with this notion, this research group found that SENP2 could desumoylates TGF-β RI receptor but not Smad4 in bladder cancer cells.

It is well documented that TGF-β promotes the EMT and metastasis in later stages of cancer progression. TGF-β induces Snail and Slug to downregulate the expression E-cadherin, a key repressor of metastasis^37^. Our findings revealed that SENP2 is involved in TGF-β-induced cell migration through the regulation of Smad4 desumoylation, correlating with the regulation of Snail, Slug and MMP9 (Figs 5A and S4). Besides, SENP2 participates in sphere formation and expression of CD44, a cell surface marker for breast cancer stem cells^38^. Our finding implicate that SENP2 not only plays an important role in TGF-β-induced cell migration but also facilitates induction of cells with CSC properties.

In sum, our data provide an additional paradigm for substrate recognition and specificity of SENP2 in alternation of substrate sumoylation. It is also noteworthy that SENP2 upregulates cell migration in breast cancer cells and also participates in cancer stemness.

## Materials and Methods

### Yeast Two-hybrid and β-Galactosidase (β-Gal) Assays

LexA-Smad4 and Gal-AD-SENP variant constructs were co-transformed into L40 yeast. After overnight incubation, the resulting yeast cells were cultured in medium lacking tryptophan and leucine for selection of diploid cells. Diploid cells were further transferred to medium lacking tryptophan, leucine, and histidine and with or without X-gal for scoring protein-protein interactions. Positive clones were selected for a subsequent assay. β-Gal liquid assays were performed with the Galacto-light Plus kit (Tropix, Bedford, MA, USA), and three separate liquid cultures for each yeast transformant were assayed.

### Cell Culture, Transient Transfection, and Luciferase Reporter Assay

HeLa, MDA-MB231, and MDA-MB-468 cell lines were obtained from American Type Culture Collection (Manassas, VA, USA). Cells were grown in Dulbecco’s modified Eagle’s medium (DMEM) supplemented with 10% fetal bovine serum (FBS). For co-immunoprecipitation (co-IP) and Western blot analyses, cells were harvested at 48 h after transfection. For the reporter gene assay, 10^5^ cells were seeded on 24-well plates 24 h prior to transfection. Various expression constructs, together with the 3TP-Lux (125 ng) or SBE4-Luc (125 ng) and TK-Renilla (25 ng) reporter plasmids (as an indicator of normalization of transfection efficiency) were introduced into these cells by a PolyJet™ *in vitro* DNA transfection reagent (SignaGen Laboratories, Rockville, MD, USA). After starvation with 0.2% FBS-containing medium for 24 h, cells were treated with 100 pm TGF-β for 24 h. Cells were lysed and assayed for relative luciferase activity with the Dual-Glo® Luciferase Assay System (Promega, Madison, WI, USA).

### Nuclear and Cytoplasmic Extraction

Cell pellets were suspended in 800 μl of hypotonic buffer (10 mM HEPES, 1.5 mM MgCl_2_, 10 mM KCl, 0.05% NP40, pH 8.0), containing 1 mM dithiothreitol (DTT) and protease inhibitor cocktail (Roche, Basel, Switzerland). Cytoplasmic extraction was obtained by centrifuging the cell lysate at 3000 rpm for 5 min. Nuclear pellets were suspended in 800 μl of nuclear extraction buffer (100 mM NaCl, 20 mM HEPES, 1% Triton X-100 in water, pH 7.4) containing protease inhibitor cocktail. Nuclear fractions were collected at 12000 rpm for 15 min.

### Immunoprecipitation (IP), Desumoylation Assay, and Western Blot Analysis

Cells were lysed in RIPA buffer containing 50 mM HEPES (pH 7.5), 1% Nonidet P-40, 0.5% sodium deoxycholate, 0.15 M NaCl, 1 mM EDTA, 0.1 mM Na_3_VO_4_, 10 mM sodium fluoride, 10 mM sodium pyrophosphate, 20 mM β-glycerophosphate disodium, 20 mM N-ethylmaleimide (NEM), and a complete protease inhibitor mixture (Roche Applied Science, Pleasanton, CA, USA). For co-IP assays of SENP2-Smad4 interactions, lysates were mixed with the indicated antibody for 2 h, and incubated with Protein A/G for an additional 2 h on a gentle rotary shaker, followed by three washes with lysis buffer. Bead-bound proteins were analyzed by western blotting as described previously^39^. For the *in vitro* desumoylation assay, 1 µg of purified SUMOylated-Smad4-Linker was mixed with 100 ng of purified GST-SENP2-C or GST-SENP2-C-C/S and incubated at 30 °C for 60 min in 20 µl of reaction buffer containing 10 mM Tris-HCl (pH 7.4), 150 mM NaCl, and 1 mM DTT. The reaction products were subjected to sodium dodecylsulfate polyacrylamide gel electrophoresis (SDS-PAGE) and Western blot analyses. For experiments of Smad4 desumoylation of cells by SENP2, 1.2 µg of tagged-Smad4 was cotransfected with 0.3 µg of green fluorescent protein (GFP)-SUMO1. Lysates were mixed with the indicated antibody for 2 h, and incubated with Anti-HA magnetic beads (88836, Thermo Scientific, Waltham, MA, USA) or Protein A/G (GE Healthcare Bio-Sciences, Pittsburgh, PA, USA) for an additional 2 h on a gentle rotary shaker, followed by washing with lysis buffer three times. Bead-bound proteins were analyzed by western blotting. The following primary antibodies were used: anti-SMAD4 (38454, Cell Signaling Technology, Danvers, MA, USA), anti-Flag (F3165, Sigma-Aldrich, St. Louis, MO, USA); anti-HA (HA.11, Covance, Princeton, NJ, USA); anti-GFP (ab13970, Abcam, Cambridge, UK); anti-SLUG (sc-166476, Santa Cruz Biotechnology, Dallas, TX, USA); anti-SENP2 (GTX111245); anti-MMP9 (GTX23159); anti-SNAI1 (GTX100754); anti-CD44 (GTX102111) anti-actin (GTX11003); and anti-His (GTX115045) (all purchased from GeneTex; Hsinchu City, Taiwan); and anti-SUMO-1 was generated by LTK BioLaboratories (Taoyuan, Taiwan).

### Far-Western Assays

^35^S-Methionine-labeled SENP2-C-CS proteins were synthesized by the TNT reticulocyte lysate system (Promega, Madison, WI, USA). Two micrograms of recombinant GST, GST-Smad4-MH1, and GST-Smad4-Linker proteins prepared from bacteria was subjected to electrophoresis on 12% SDS-PAGE in duplicate. One copy was subjected to Coomassie blue staining for the protein loading control, and the other one was transferred to a nitrocellulose membrane. The membrane was blocked with PBST (phosphate-buffered saline and 0.1% Tween 20) which contained 5% milk for 2 h, and then incubated with isotope-labeled SENP2-C-CS for 4 h. After being washed with PBST three times, the sample was analyzed by autoradiography.

### Establishment of HeLa Short Hairpin (sh)SENP2 Stable Cells and MDA-MB-231 SENP2 Gene-knockout Stable Cells

The SENP2 expression cDNA clone were ligated with PCR amplified fragment of Homo sapiens SENP2 (accession number: NM_021627). For knockdown experiments, HeLa cells were transfected with shRNA for the 3′ untranslated region (UTR) of SENP2 (TRCN0000004579; target sequence: CCACACAAGAACAAACGCTAA) and selected with puromycin (5 µg/ml) for 2 weeks. For MDA-MB-231-2B and SENP2 gene-knockout stable cells, MDA-MB-231 cells were transfected with pSpCas9 (BB)-Puro-sgRNA-2B (target sequence: TTTCTGCGACCGGTCGGTG) and selected with puromycin (1 µg/ml). After cells expanded from single cell, genomic DNA was isolated with the EasyPrep Genomic DNA Extraction Kit (Tools, New Taipei City, Taiwan) and subjected to a T7E1 assay. Exon 1 DNA fragments were amplified by a polymerase chain reaction (PCR) with T7E1 assay primers (forward (F): 5′-CTGACGAGATCGGAAGGG-3′ and reverse (R): 5′-CTTTGATTCAGCCAGGCTCAC-3′) and purified by a Qiagen PCR purification kit (Hilden, Germany). Purified DNA was subjected to re-hybridization (hetero-duplex synthesis with the following mixture: 400 ng purified DNA, 2 μl NEB buffer 2, and double-distilled (dd)H_2_O (the total volume of the mix was 20 μl), and then the mixture was heated to 37 °C for 30 min and 95 °C for 5 min) and the T7E1 assay was run (NEB Cat#M0302S). Finally, the resultant mixtures were analyzed by gel electrophoresis with a 1.8% gel.

### Real-time Quantitative (q)PCR

Total cellular RNAs from MDA-MB-231 cells depleted of SENP2 by CRISPR or transfected with GFP-tagged SENP2 or mutants were extracted using the TRIzol reagent (Invitrogen, Carlsbad, CA. USA). RNA (5 μg) of each sample was then reverse-transcribed using the ThermoScript reverse transcription-PCR system (Invitrogen) in a 20-μl reaction mix. The reverse-transcription reaction product (250 ng) was used for the real-time qPCR with the SYBR™ Green assay system for SENP2 (F: 5′-GCGGCTAGCGACGATCTCCTTGAACTTACA-3′ and R: 5′-GCGGCTAGCGCTCCAATGTACCTTCCGATG-3′), *Snai1* (F: 5′-CCCTCAAGATGCACATCCGAA-3′ and R: 5′-GACTCTTGGTGCTTGTGGAGCA-3′), *Slug* (F: 5′-AGATGCATATTCGGACCCAC-3′ and R: 5′-CCTCATGTTTGTGCAGGAGA-3′), *MMP-9* (F: 5′-GAGTGGCAGGGGGAAGATGC-3′ and R: 5′-CCTCAGGGCACTGCAGGATG-3′), and hGAPDH (F: 5′-GCACCGTCAAGGGCTGAGAAC-3′ and R: 5′-TGGTGAAGACGCCAGTGGA-3′) which utilized the Applied Biosystems PRISM 7300 Real-Time PCR System, according the manufacturer’s protocol. For each sample, the average threshold (Ct) value was determined from triplicate assays, and the ΔCt value was determined by subtracting the average *hGAPDH* Ct value from the average *SENP2*, *Snai1, Slug*, or *MMP9* Ct value.

### Cell Migration and Invasion Assay

The invasive and migration behavior of transfected cells (2~6 × 10^4^) were washed with 1 × PBS, resuspended in DMEM with 0.2% FBS, and added to the cell culture inserts (24 wells; 8 μm pore, BD Sciences, San Jose, CA). For invasion assay, cell culture inserts were coated with 100 μl of 1:10 Matrigel/Medium dilutions (BD Sciences) and allowed to solidify at 37 °C for 1 h before seeding cells. The medium in the lower compartment was supplemented with TGF-β or 10% FBS. After 16 h, the insert was carefully taken out, fixed by 5% glutaraldehyde for 10 min, and stained with crystal violet. The number of migrating and invasive cells was counted under a microscope. Different views were randomly chosen, and averages were counted.

### Cell viability assay

MDA-MB231-2B or HeLa-shSENP2 cells transfected with 3xFlag-SENP2 or SENP2-DM were seeding in 96 well plate and starvation in DMEM with 0.2% FBS for 24 h followed by treated with or without 100 ng/ml TGF-β for indicated times. Cell viability was determined by adding AlamarBlue reagent (Invitrogen) as 10% of the medium volume followed by a 1 h incubation at 37 °C. The fluorescence is read by a fluorescence excitation wavelength of 560 nm and emission at 590 nm using a fluorescence spectrophotometer (TECAN-Infinite® M1000 PRO).

### Sphere Cell Formation Assay

Cells at 10^5^ were washed with 1 × PBS and suspended in MEGM, including DMEM/F-12 (Invitrogen 11330), B27 (50x, Invitrogen 17504-044), Epidermal growth factor (EGF) (10 μg/ml, Sigma E9644; St. Louis, MO, USA), insulin (10 mg/ml, Sigma I9275), hydrocortisone (0.4 mg/ml, Sigma H0888), basic fibroblast growth factor (bFGF; 20 μg/ml, Sigma F0291), and heparin (8 mg/ml, Sigma). We seeded cells in a low attachment culture dish, then added MEGM to 10 ml, and incubated the mixture at 37 °C with 5% CO_2_. After a week, the number of sphere cells was counted under a microscope. Different views were randomly chosen, and averages were counted. Each sphere cell formation experiment was performed in triplicate.

### Statistical Analysis

Data processing and statistical analyses were performed with SAS 9.4 (SAS Institute, Cary, NC, USA) and the GraphPad Prism 6 statistical software package. Student’s *t*-tests were used to determine the significance of differences between samples as indicated in figures. *p* values of <0.05 were considered statistically significant.

## Electronic supplementary material

Supplementary data
