# Author Correction: The role of sentrin-specific protease 2 substrate recognition in TGF-β-induced tumorigenesis

**DOI:** 10.1038/s41598-020-66542-4

**Published:** 2020-06-04

**Authors:** Che-Chang Chang, Yen-Sung Huang, Ying-Mei Lin, Chia-Ju Lin, Jen-Chong Jeng, Shin-Mei Liu, Tsai-Ling Ho, Ruei-Ting Chang, Chun A. Changou, Chun-Chen Ho, Hsiu-Ming Shih

**Affiliations:** 1 Graduate Institute of Translational Medicine, College of Medical Science and Technology, Taipei Medical University, Taipei, 11031 Taiwan; 2 The Ph.D. Program for Translational Medicine, College of Medical Science and Technology, Taipei Medical University, Taipei, 11031 Taiwan; 3 Ph.D Program in Biotechnology Research and Development, College of Pharmacy, Taipei Medical University, Taipei, 11031 Taiwan; 4 Traditional Herbal Medicine Research Center of Taipei Medical University Hospital, Taipei, 11031 Taiwan; 5 Institute of Biomedical Sciences, Academia Sinica, Taipei, 11529 Taiwan; 6 The Ph.D. Program for Cancer Biology and Drug Discovery, College of Medical Science and Technology, Taipei Medical University, Taipei, 11031 Taiwan; 7 Institute of Molecular and Genomic Medicine, National Health Research Institutes, Miaoli County, 35053 Taiwan

Correction to: *Scientific Reports* 10.1038/s41598-018-28103-8, published online 28 June 2018

This Article contains an error in Figure 4A. The image for the ShSENP2 + GFP-SENP2-DM condition is incorrect. The correct Figure 4A appears below as Figure 1.

**Figure 1:**
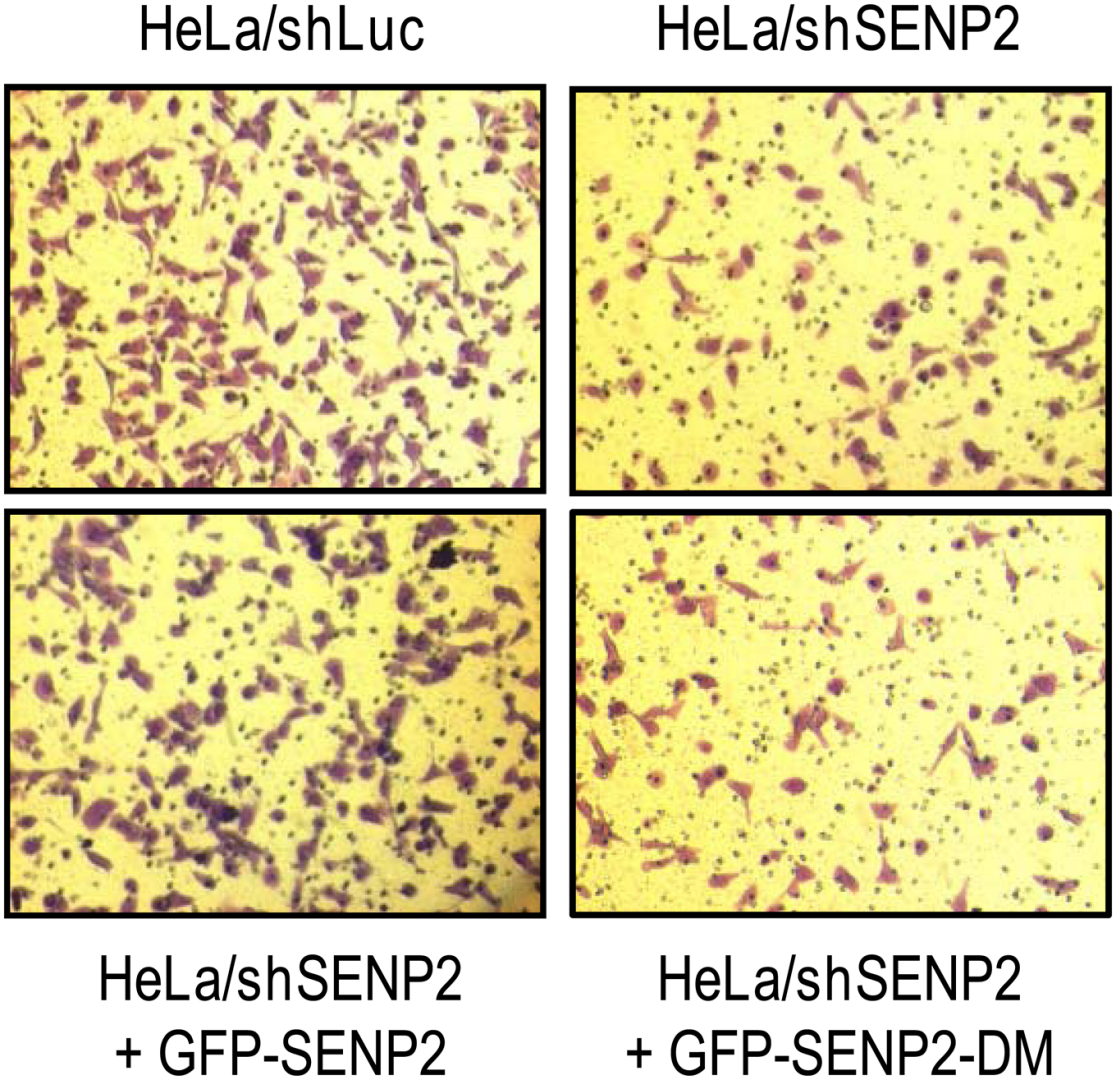
.

