# Supplementary material for: The role of sentrin-specific protease 2 substrate recognition in TGF-β-induced tumorigenesis
Source: Sci Rep. 2018 Jun 28;8:9786. doi: 10.1038/s41598-018-28103-8 (PMC6023881; doi:10.1038/s41598-018-28103-8)
Supplement: Supplementary file 1 — Supplementary data [file 41598_2018_28103_MOESM1_ESM.pdf]

# Supplementary information for

## The role of sentrin-specific protease 2 substrate recognition in TGF-β-induced tumorigenesis

Che-Chang Chang[1,2,3,4*], Yen-Sung Huang[5], Ying-Mei Lin[5], Chia-Ju Lin[1], Jen-Chong Jeng[5], Shin-Mei Liu[1], Tsai-Ling Ho[1], Ruei-Ting Chang[1], Chun A. Changou[2,6], Chun-Chen Ho[5], and Hsiu-Ming Shih[1,2,5,7 *]

[1] Graduate Institute of Translational Medicine, College of Medical Science and Technology, Taipei Medical University, Taipei 11031, Taiwan

[2] The Ph.D. Program for Translational Medicine, College of Medical Science and Technology, Taipei Medical University, Taipei 11031, Taiwan

[3] Ph.D Program in Biotechnology Research and Development, College of Pharmacy, Taipei Medical University, Taipei 11031, Taiwan

[4] Traditional Herbal Medicine Research Center of Taipei Medical University Hospital, Taipei 11031, Taiwan

[5] Institute of Biomedical Sciences, Academia Sinica, Taipei 11529, Taiwan

[6] The Ph.D. Program for Cancer Biology and Drug Discovery, College of Medical Science and Technology, Taipei Medical University, Taipei 11031, Taiwan

[7] Institute of Molecular and Genomic Medicine, National Health Research Institutes, Miaoli County 35053, Taiwan

* Co-corresponding authors:

Dr. Che-Chang Chang, Graduate Institute of Translational Medicine, College of Medical Science and Technology, Taipei Medical University, 250 Wu-Xing Street, Taipei 11031, Taiwan. E-mail: ccchang168@tmu.edu.tw; Tel: 886-2-27361661 ext.7630; Fax: 886-2-6638-7537

Dr. Hsiu-Ming Shih, Institute of Biomedical Sciences, Academia Sinica, 128 Academic Road, Sec. 2, Taipei 11529, Taiwan. E-mail: hmshih@ibms.sinica.edu.tw or hmshih@nhri.org.tw; Tel: 886-2-2652-3520; Fax: 886-2-2782-7654

## This supplementary files including supplementary figures and figure legends

Figure S1. SENP2 potentates TGF-β signaling via desumoylation of Smad4.

Figure S2. The SENP2$^{363\sim400}$ is crucial for interaction with Smad4 and potentiation of TGF-β-induced transcriptional activation.

Figure S3. The cell viability of SNEP2 re-introduced cells is similar to SENP2-DM re-introduced cells.

Figure S4. SENP2$^{363\sim400}$ is crucial for MMP9 expression.

Figure S5. Survival analysis of breast cancer patients.

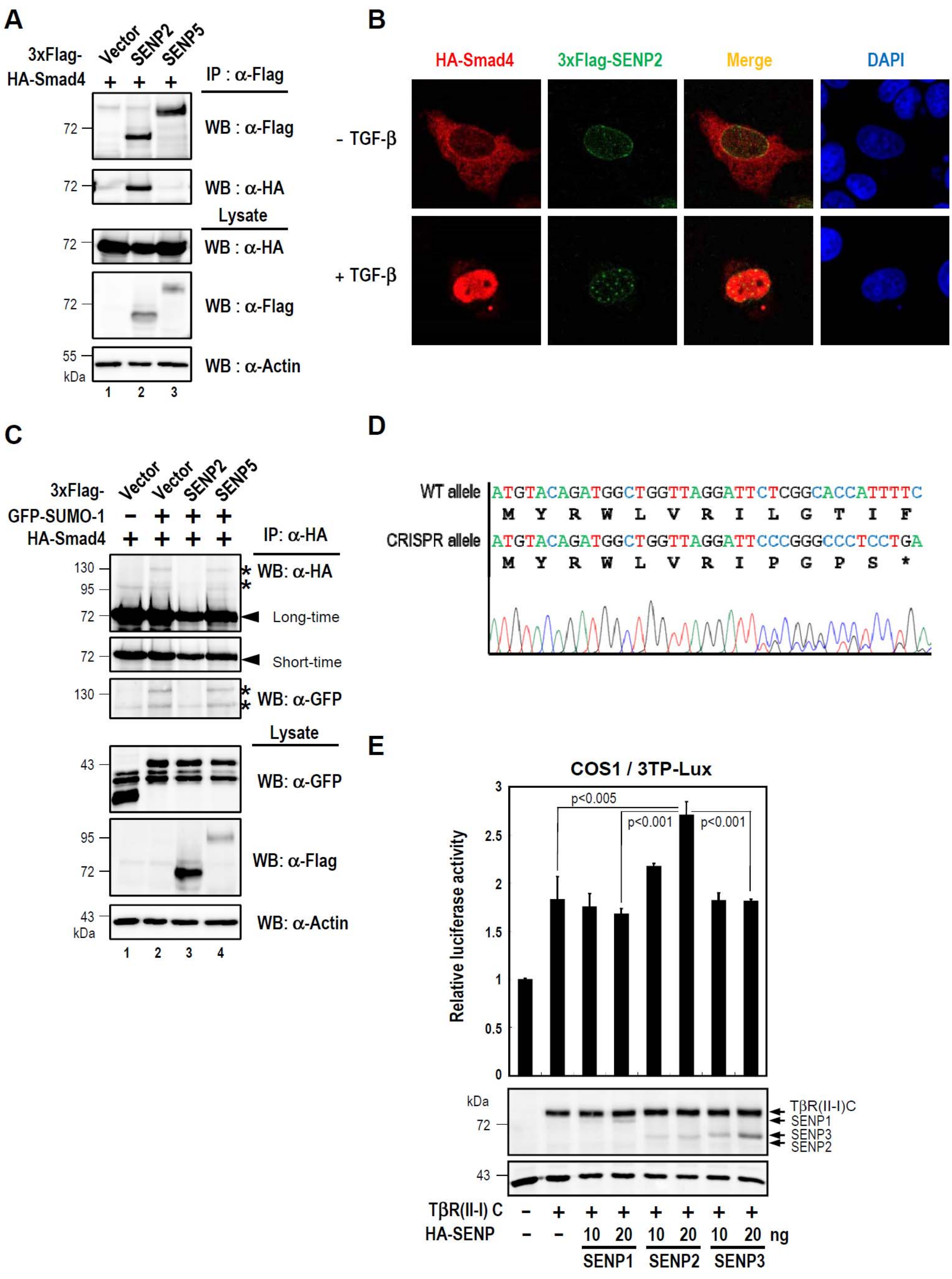
A
3xFlag-
Vector
SENP2
SENP5
HA-Smad4
IP : α-Flag
WB : α-Flag
WB : α-HA
Lysate
WB : α-HA
WB : α-Flag
WB : α-Actin
72
55
kDa
1
2
3
B
HA-Smad4
3xFlag-SENP2
Merge
DAPI
- TGF-β
+ TGF-β
C
3xFlag-
Vector
Vector
SENP2
SENP5
GFP-SUMO-1
HA-Smad4
IP: α-HA
WB: α-HA
Long-time
Short-time
WB: α-GFP
Lysate
WB: α-GFP
WB: α-Flag
WB: α-Actin
130
95
72
43
kDa
1
2
3
4
D
WT allele
ATGTACAGATGGCTGGTTAGGATTCTCGGCACCATTTTC
M Y R W L V R I L G T I F
CRISPR allele
ATGTACAGATGGCTGGTTAGGATTCCCGGGCCCTCCTGA
M Y R W L V R I P G P S *
E
COS1 / 3TP-Lux
Relative luciferase activity
p<0.005
p<0.001
p<0.001
3
2.5
2
1.5
1
0.5
0
kDa
72
43
TβR(II-I)C
SENP1
SENP3
SENP2
TβR(II-I) C
HA-SENP
10
20
ng
SENP1
SENP2
SENP3

**Figure S1. SENP2 potentates TGF-β signaling via desumoylation of Smad4.** **(A)** *Smad4 interacts with SENP2, but not SENP5.* HeLa cells were transfected with indicated plasmid constructs for 48 hours, and then harvested for analysis by immunoprecipitation (IP) and Western blot (WB) using anti-HA and anti-Flag antibodies. The expression levels of transfected proteins were analyzed by immunoblotting with indicated antibody. **(B)** *TGF-β treatment facilities the SENP2/Smad4 interaction.* HeLa cells were transfected with plasmid expressing HA-Smad4 and 3xFlag-SENP2. Transfectants were starved for 24 h, treated with or without 100 pM TGF-β for 2 h and then immunostained with anti-HA (red) and anti-Flag (green) antibodies and followed by 4',6'-diamidino-2-phenylindole staining (DAPI, blue). The cells were examined by confocal microscopy (TCS SP5 Confocal Spectral Microscope Imaging System). The overlay of the green and red images was shown (merge). **(C)** *SENP2 desumoylated Smad4, but not SENP5.* HeLa cells were transfected with expression vector for 1.5 μg of HA-Smad4 and 0.5 μg of GFP-SUMO-1, together with expression plasmids for 3xFlag-SENP2 or SENP5 as indicated. The transfected cells were lysed in RIPA buffer in the presence of 20 mM NEM, and subjected to immunoprecipitation with anti-HA antibody. Western blots analysis with anti-HA and anti-GFP antibodies show sumoylation levels of HA-tagged Smad4. The expression levels of GFP-SUMO-1 and 3xFlag-SENPs were determined by WB analysis. *Asterisk* and *arrowhead*, SUMO-1-modified and -unmodified Smad4 proteins, respectively. **(D)** *The verification of CRISPR-generated SENP2 knock out MDA-MB231 cell.* The sequence of MDA-MB-231-2B, SENP2 gene-knockout stable cells. Exon 1 DNA fragments were amplified with Pfu polymerase and T7E1 assay primers (F: 5'-CTGACGAGATCGGAAGGG-3' R: 5'-CTTTGATTCAGCCAGGCTCAC-3') by a PCR. After being cloned into the pHE vector (Tools, New Taipei City, Taiwan), plasmid DNA was analyzed. **(E)** *SENP2 activates TGF-β-induced transcriptional potential.* Reporter gene analysis of COS-1 cells transfected with a constitutively active chimera of the TGF-β receptor and 3TP-Lux reporter constructs alone or with SENPs. Error bars show standard deviations from three experiments performed in triplicate. Statistical significance was ascertained with Student's *t*-test.

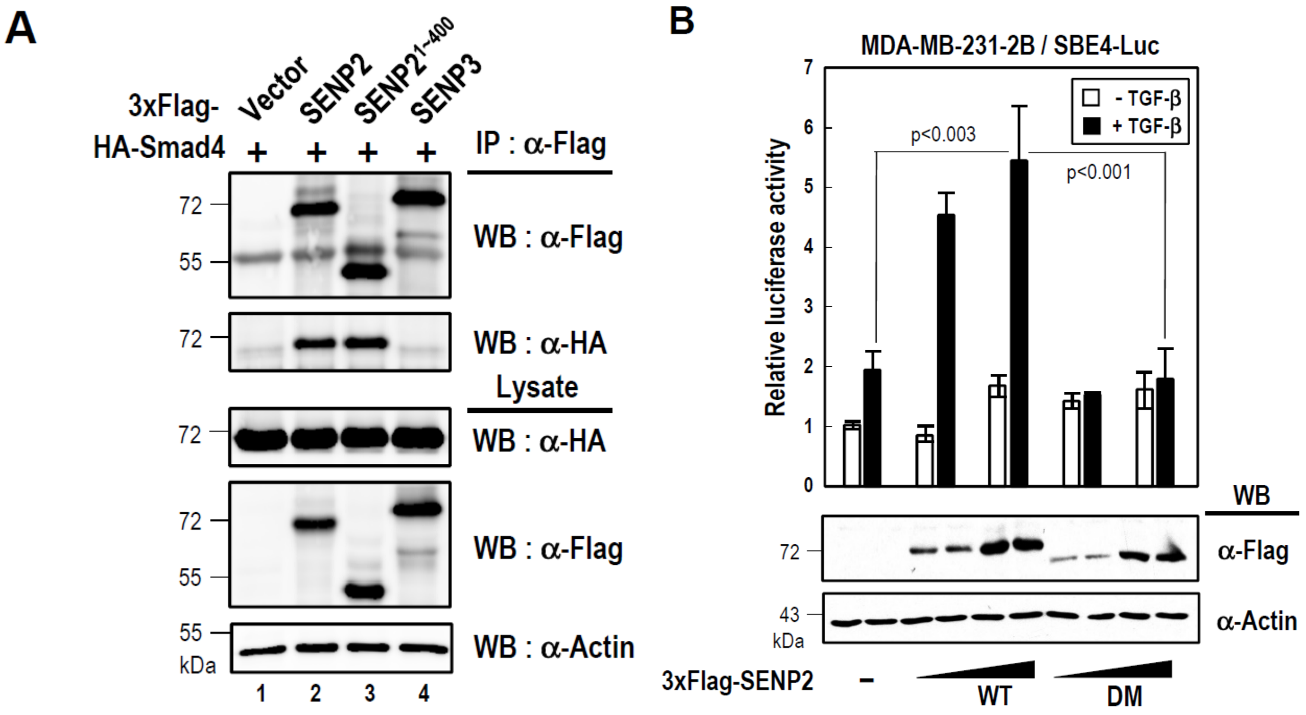


**Figure S2. The SENP2$^{363\sim400}$ is crucial for interaction with Smad4 and potentiation of TGF-β-induced transcriptional activation. (A)** SENP2$^{1\sim400}$ is sufficient for Smad4 interaction. HeLa cells were transfected with indicated plasmid constructs for 48 hours, and then harvested for analysis by immunoprecipitation (IP) and Western blot (WB) using anti-Flag and anti-HA antibodies. The expression levels of transfected proteins were measured by immunoblotting with indicated antibody. SENP3 was as negative control for IP. (B) *SENP2-DM loss the activation ability to TGF-β-induced transcriptional potential.* MDA-MB-231-2B stable cells were transfected with the SBE4-Luc and TK-Renilla reporter construct with 3xFlag-SENP2 or SENP2-DM plasmids as indicated. After transfection, the cells were starved for 24 h followed by TGF-β treatment for 24 h. The cells were harvested and subjected to reporter assays as described in “Material and Method”. Relative luciferase activity is represented as the means ± S.D. from three independent experiments. Statistical significance was ascertained with Student’s *t*-test.

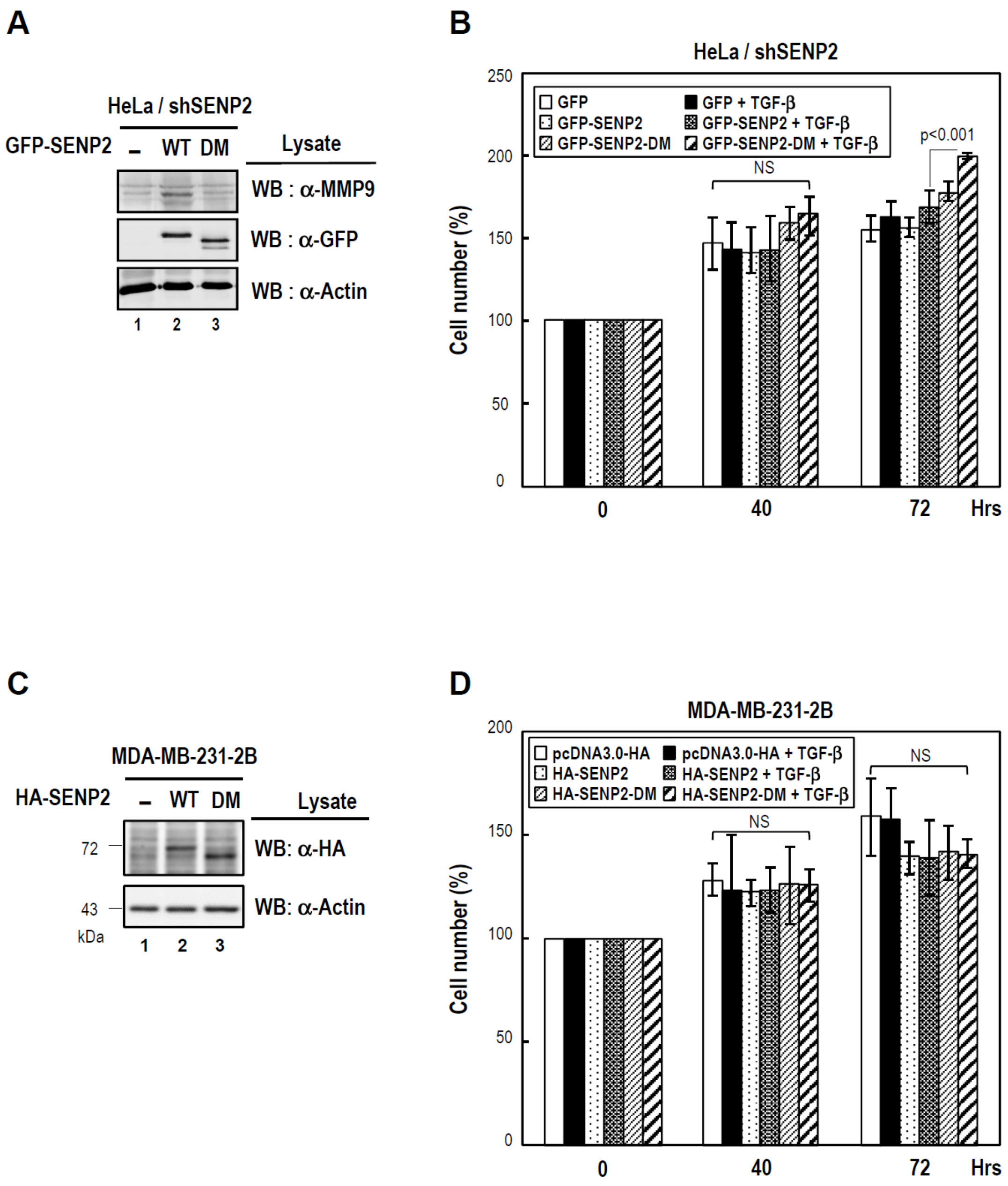


**Figure S3. The cell viability of SNEP2 re-introduced cells is similar to SENP2-DM re-introduced cells. (A and C)** *The expression level of SENP2 and SENP2-DM in SENP2 knockdown cells.* The shSENP2 Hela or MDA-MB-231-2B cells were transfected with expression plasmid as indicated. The expression levels of SENP2 and SENP2-DM were measured by immunoblotting with indicated antibody. **(B and D)** *The cell proliferation of SENP2 and SENP2-DM re-introduced cells.* 2x10$^3$ cells from (A and C) were seeding in 96 well plate and starved for 24hr followed by treated with or without 100 ng/ml TGF-β for indicated times. Cell viability was determined as described in “Material and Method”. Statistical significance was ascertained with Student’s *t*-test.

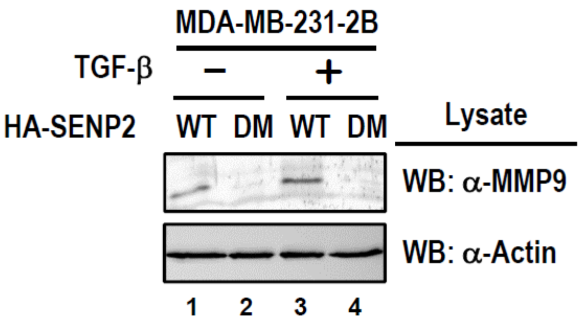


**Figure S4. SENP2$^{363\sim400}$ is crucial for MMP9 expression.** Western blots show protein levels of MMP9 in HA-SENP2 variant-reintroduced MDA-MB231-2B stable cells. Cells were starved for 24 h and with or without TGF-β treatment.

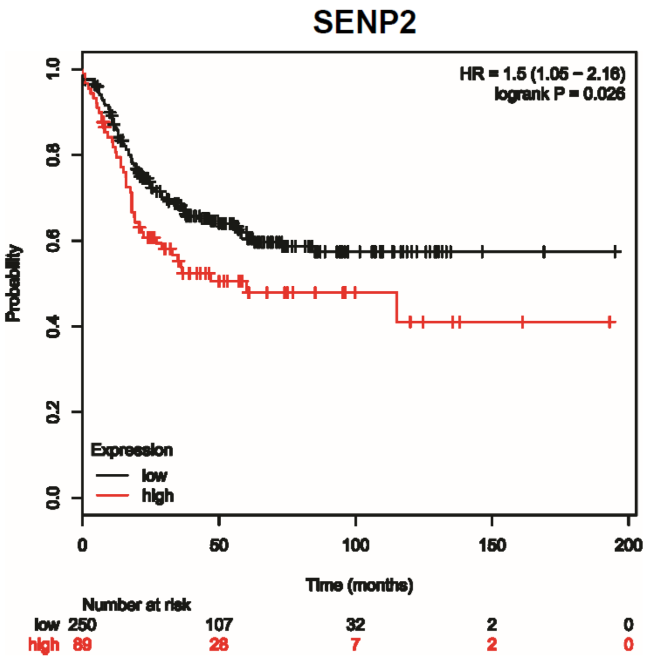


**Figure S5. Survival analysis of breast cancer patients.** For the survival analysis, relapse-free survival (RFS)-stratified by expression of the SENP2 gene is presented as Kaplan-Meier plots. Breast cancer patients were divided into two groups based on the median expression level of the gene of interest, and plotted data were downloaded from www.kmplot.com (2014 release version, Sept. 2015 download date). *p* values and survival analyses were calculated based on the log-rank statistical method and are presented as the Kaplan-Meier plots generated.

Fig. 1B

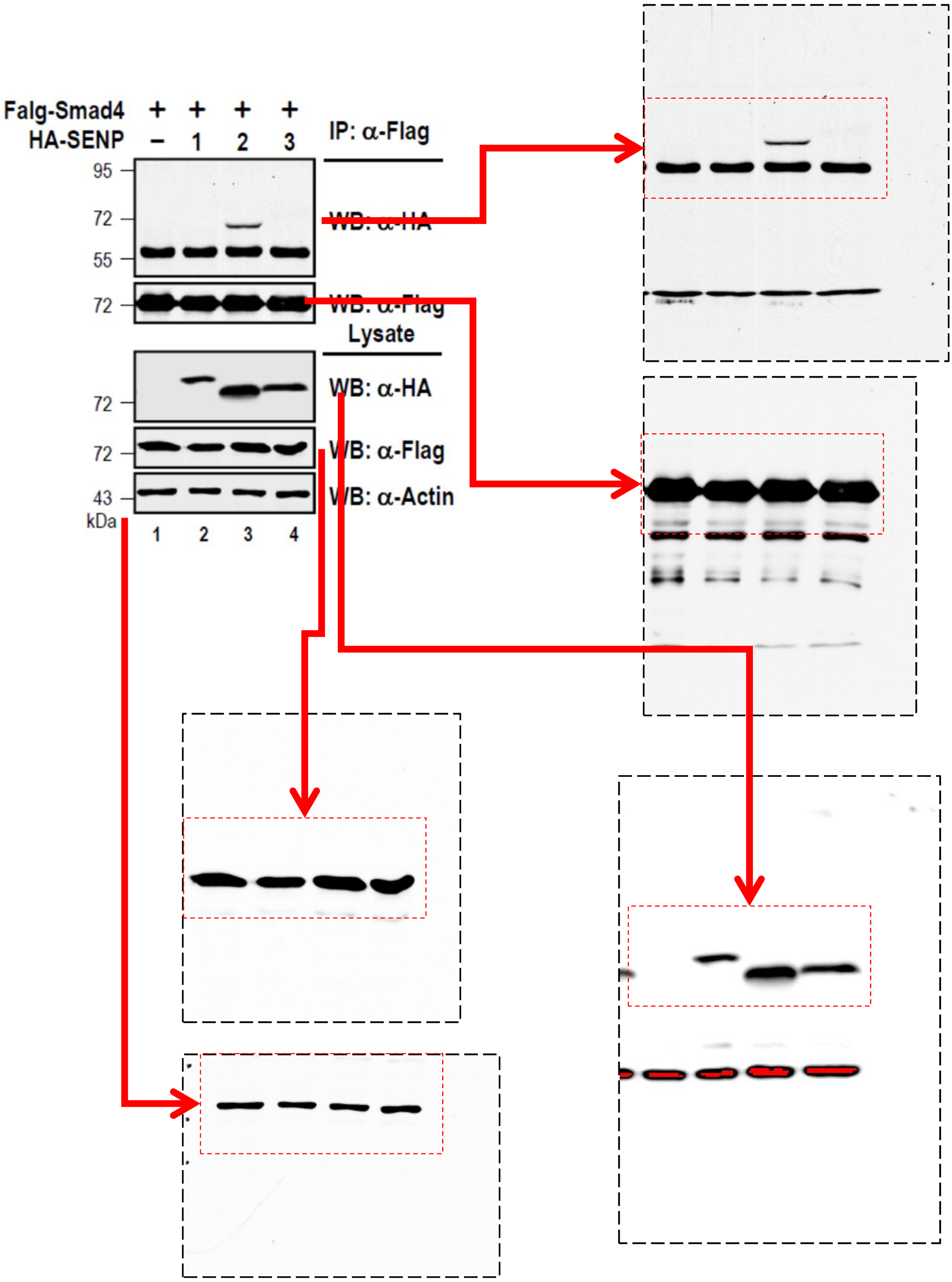

Falg-Smad4 + + + +
HA-SENP – 1 2 3
IP: α-Flag
95
72
55
WB: α-HA
72
WB: α-Flag
Lysate
WB: α-HA
72
72
WB: α-Flag
43
WB: α-Actin
kDa
1 2 3 4

Fig. 1C

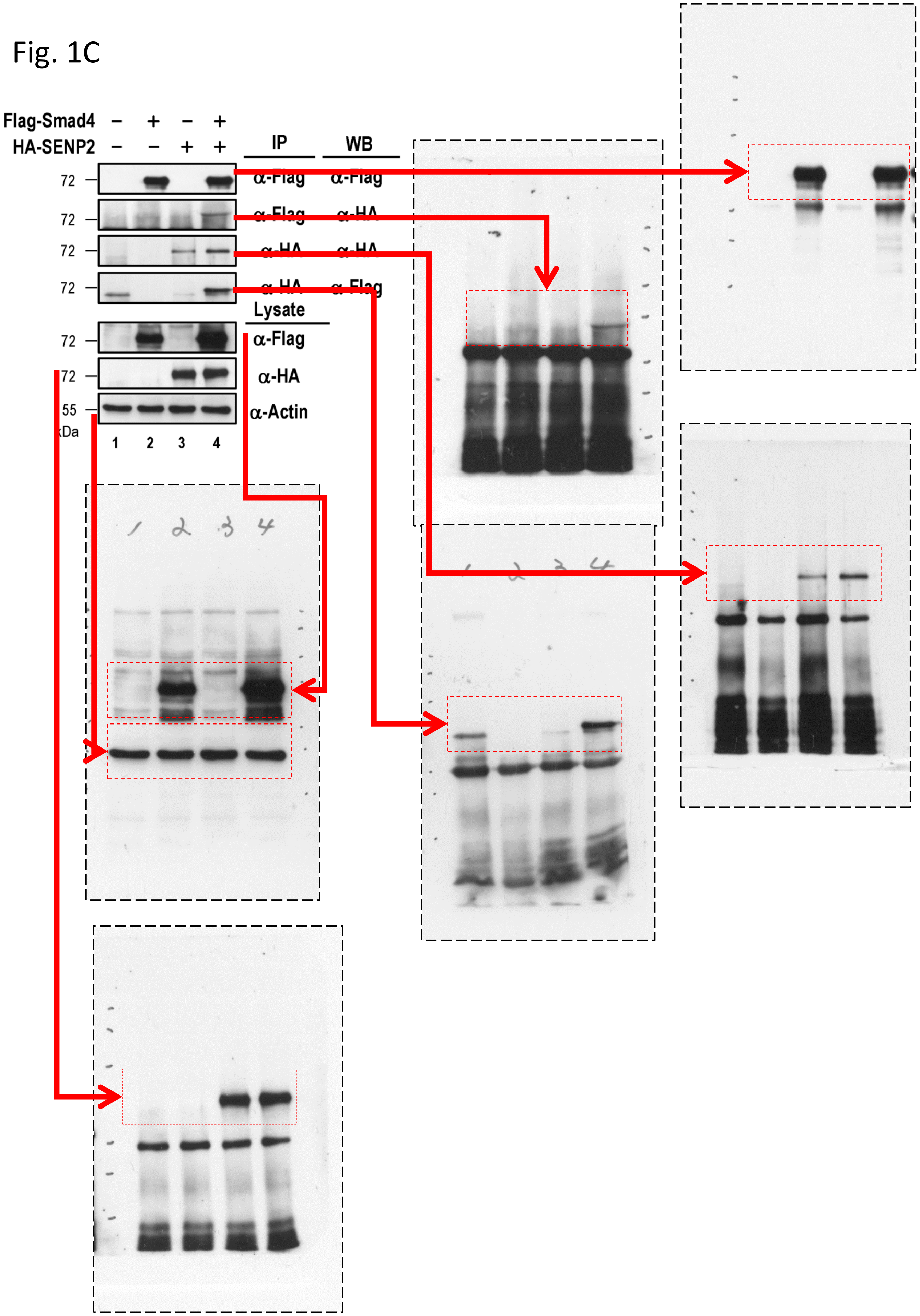
Flag-Smad4 – + – +
HA-SENP2 – – + +
IP
WB
α-Flag α-Flag
α-Flag α-HA
α-HA α-HA
α-HA α-Flag
Lysate
α-Flag
α-HA
α-Actin
72
55
kDa
1 2 3 4

Fig. 1D

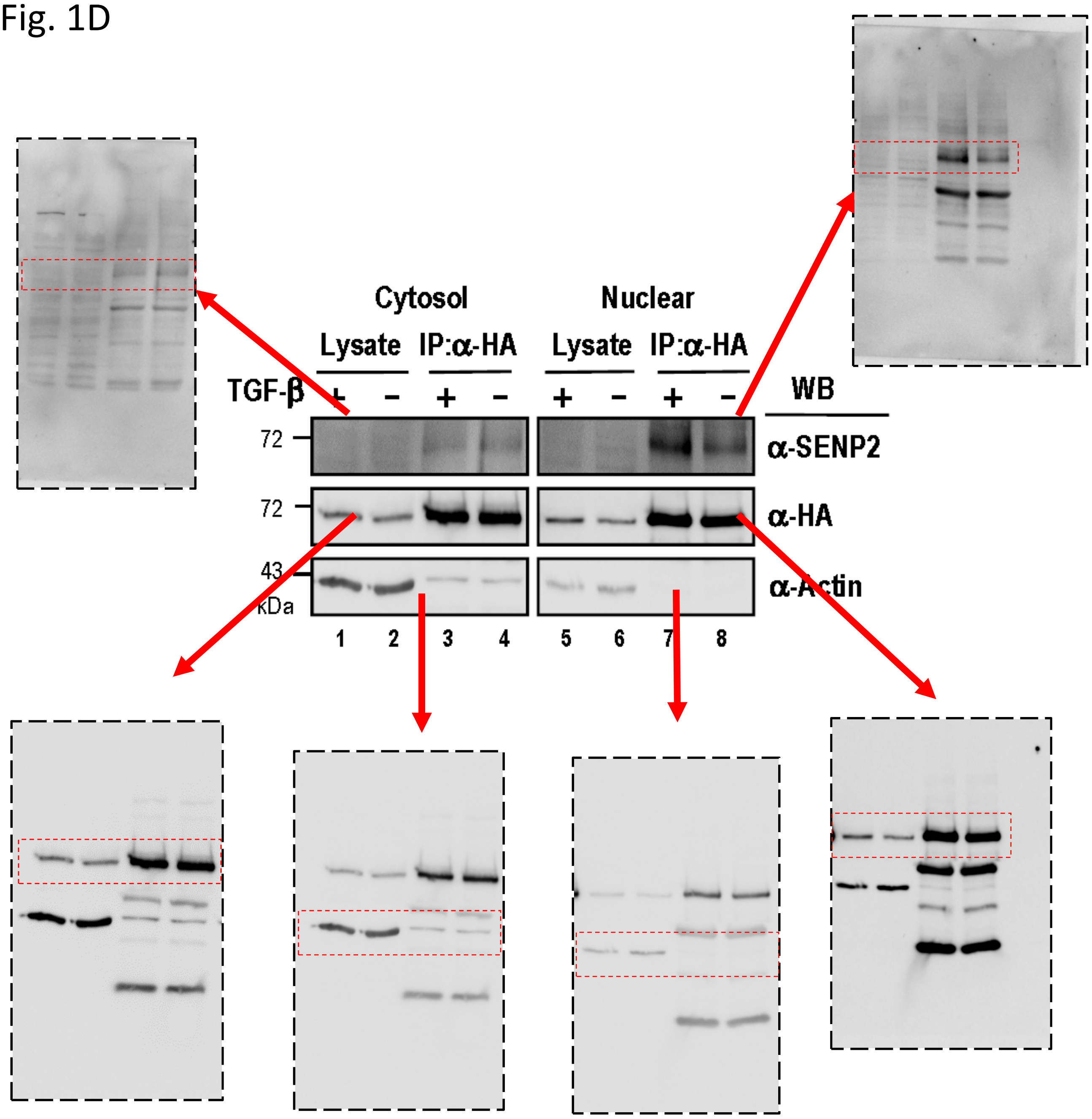

Cytosol
Nuclear
Lysate
IP:α-HA
Lysate
IP:α-HA
TGF-β
+
–
+
–
+
–
+
–
WB
72
α-SENP2
72
α-HA
43
kDa
α-Actin
1
2
3
4
5
6
7
8

Fig. 1E

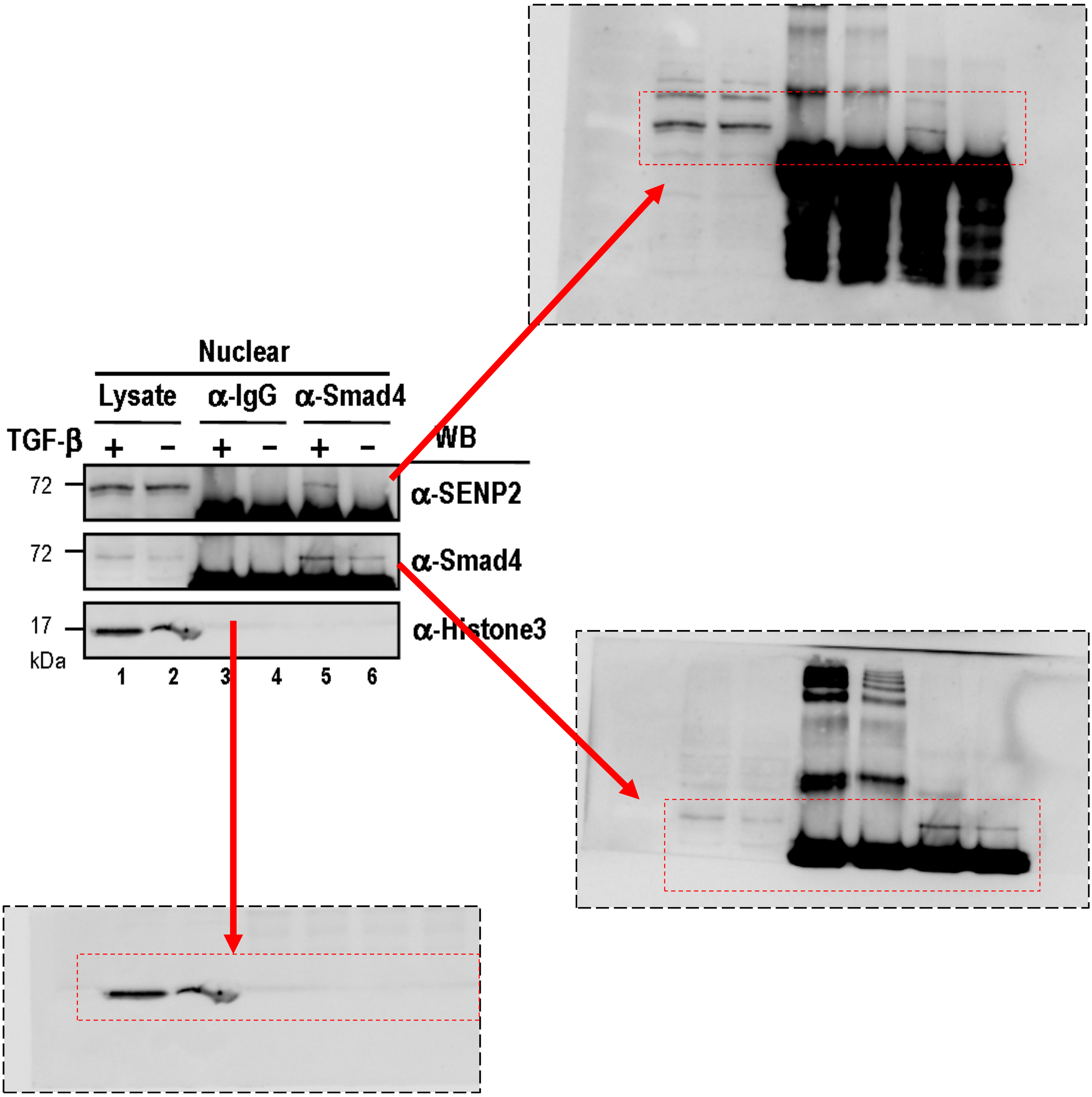

Nuclear
Lysate
α-IgG
α-Smad4
TGF-β
+
–
+
–
+
–
WB
72
α-SENP2
72
α-Smad4
17
α-Histone3
kDa
1
2
3
4
5
6

Fig. 1F

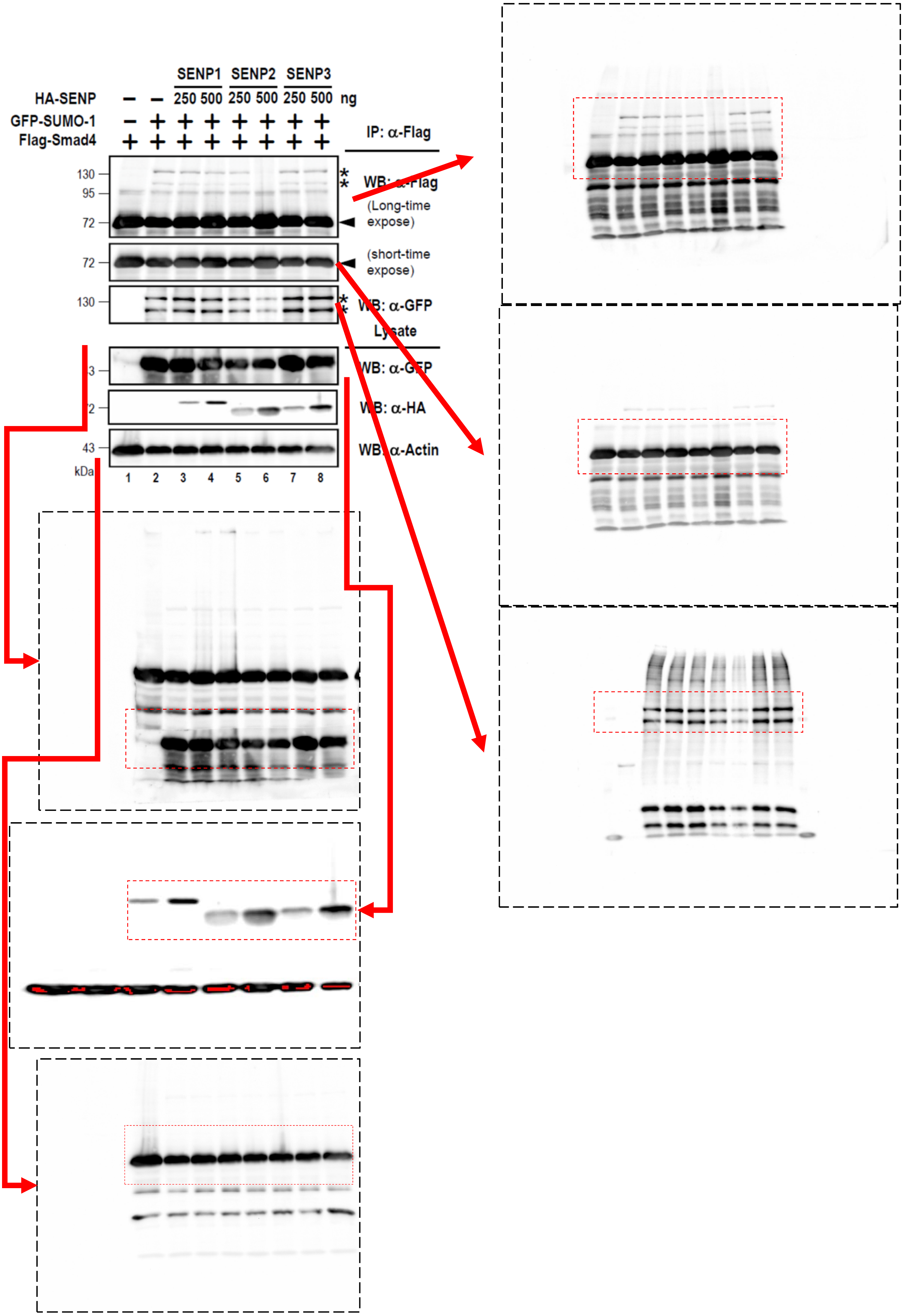

SENP1 SENP2 SENP3
HA-SENP – – 250 500 250 500 250 500 ng
GFP-SUMO-1 – + + + + + + +
Flag-Smad4 + + + + + + + +
IP: α-Flag
130
95
72
WB: α-Flag
(Long-time expose)
72
(short-time expose)
130
WB: α-GFP
Lysate
WB: α-GFP
WB: α-HA
43
WB: α-Actin
kDa
1 2 3 4 5 6 7 8

Fig. 1G

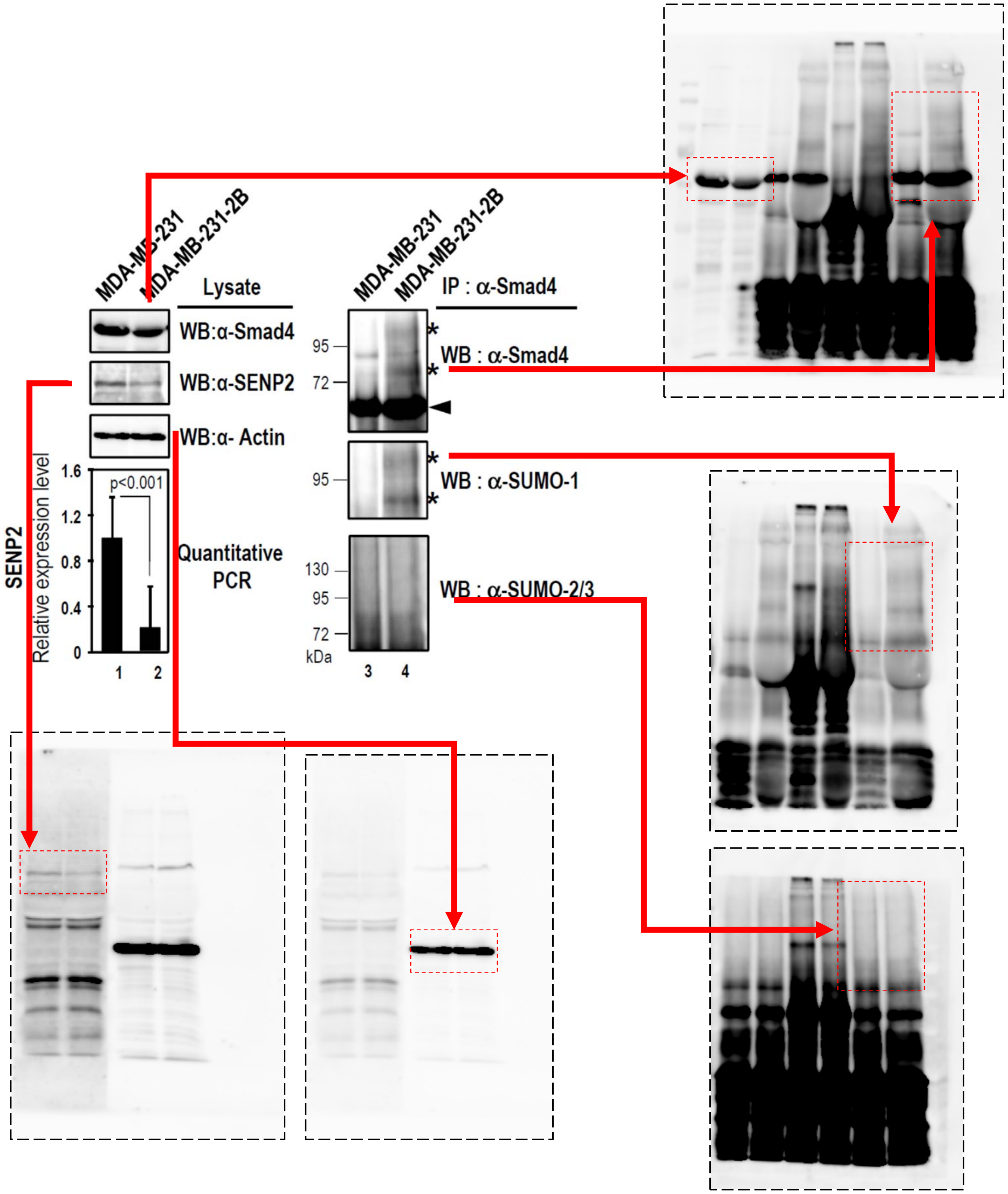

MDA-MB-231
MDA-MB-231-2B
Lysate
WB:α-Smad4
WB:α-SENP2
WB:α- Actin
SENP2
Relative expression level
p<0.001
1.6
1.2
0.8
0.4
0
1
2
Quantitative PCR
MDA-MB-231
MDA-MB-231-2B
IP : α-Smad4
WB : α-Smad4
WB : α-SUMO-1
WB : α-SUMO-2/3
95
72
130
kDa
3
4

Fig. 1H

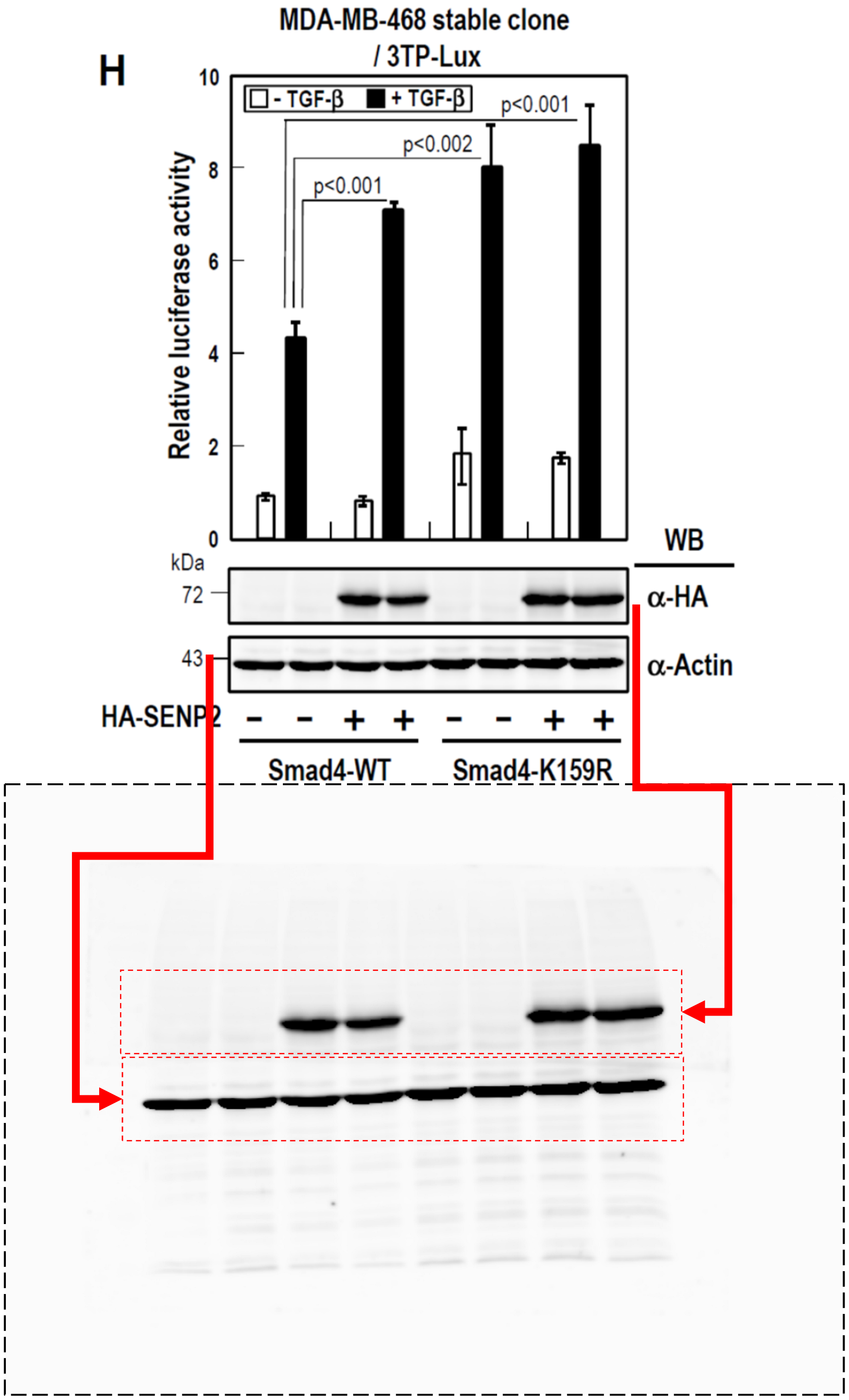

H
MDA-MB-468 stable clone
/ 3TP-Lux
- TGF-β
+ TGF-β
p<0.001
p<0.002
p<0.001
Relative luciferase activity
10
8
6
4
2
0
WB
kDa
72
43
α-HA
α-Actin
HA-SENP2
– – + + – – + +
Smad4-WT
Smad4-K159R

Fig. 2C

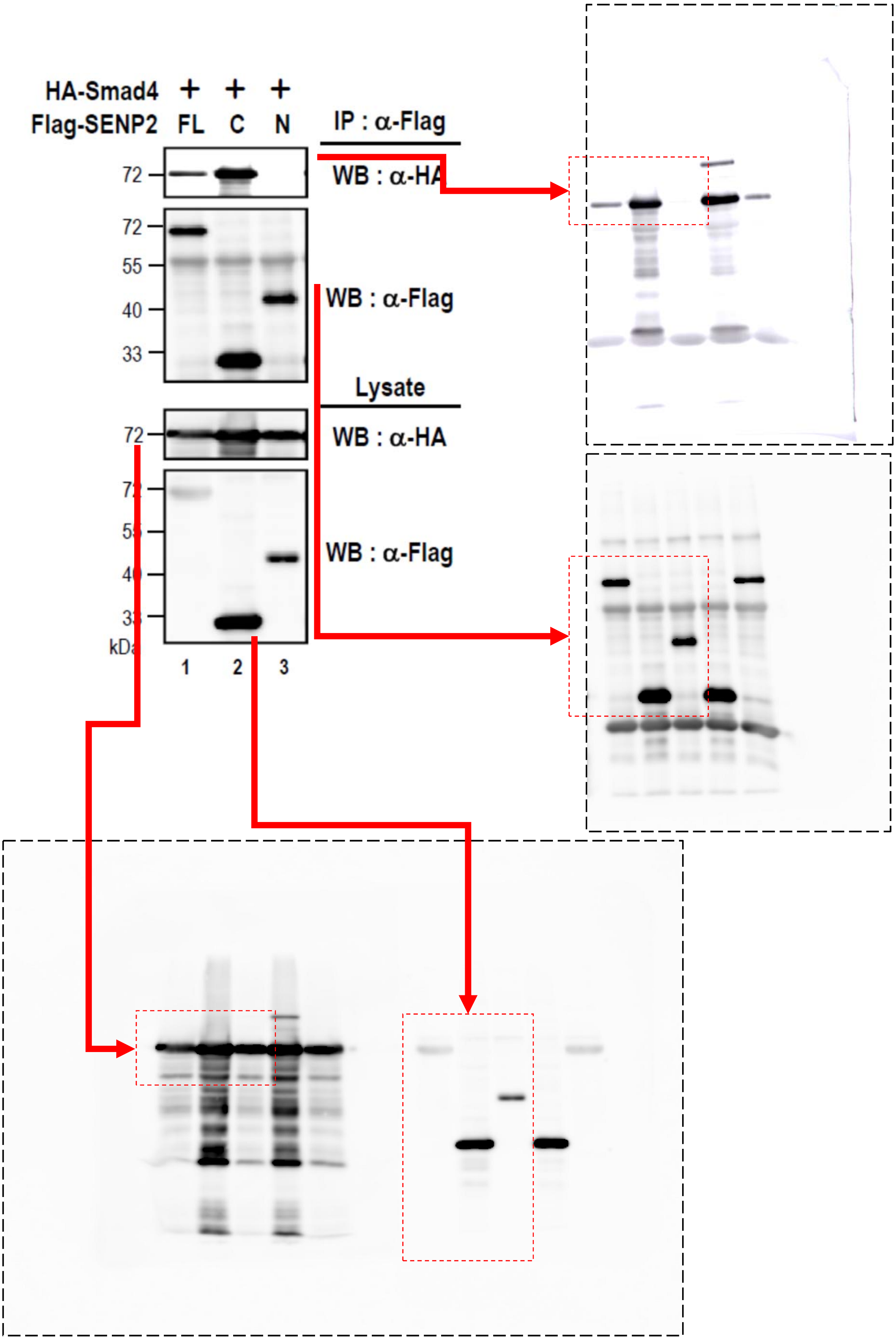

HA-Smad4 + + +
Flag-SENP2 FL C N
IP : α-Flag
WB : α-HA
WB : α-Flag
Lysate
WB : α-HA
WB : α-Flag
72
72
55
40
33
72
kDa
1 2 3

Fig. 2D

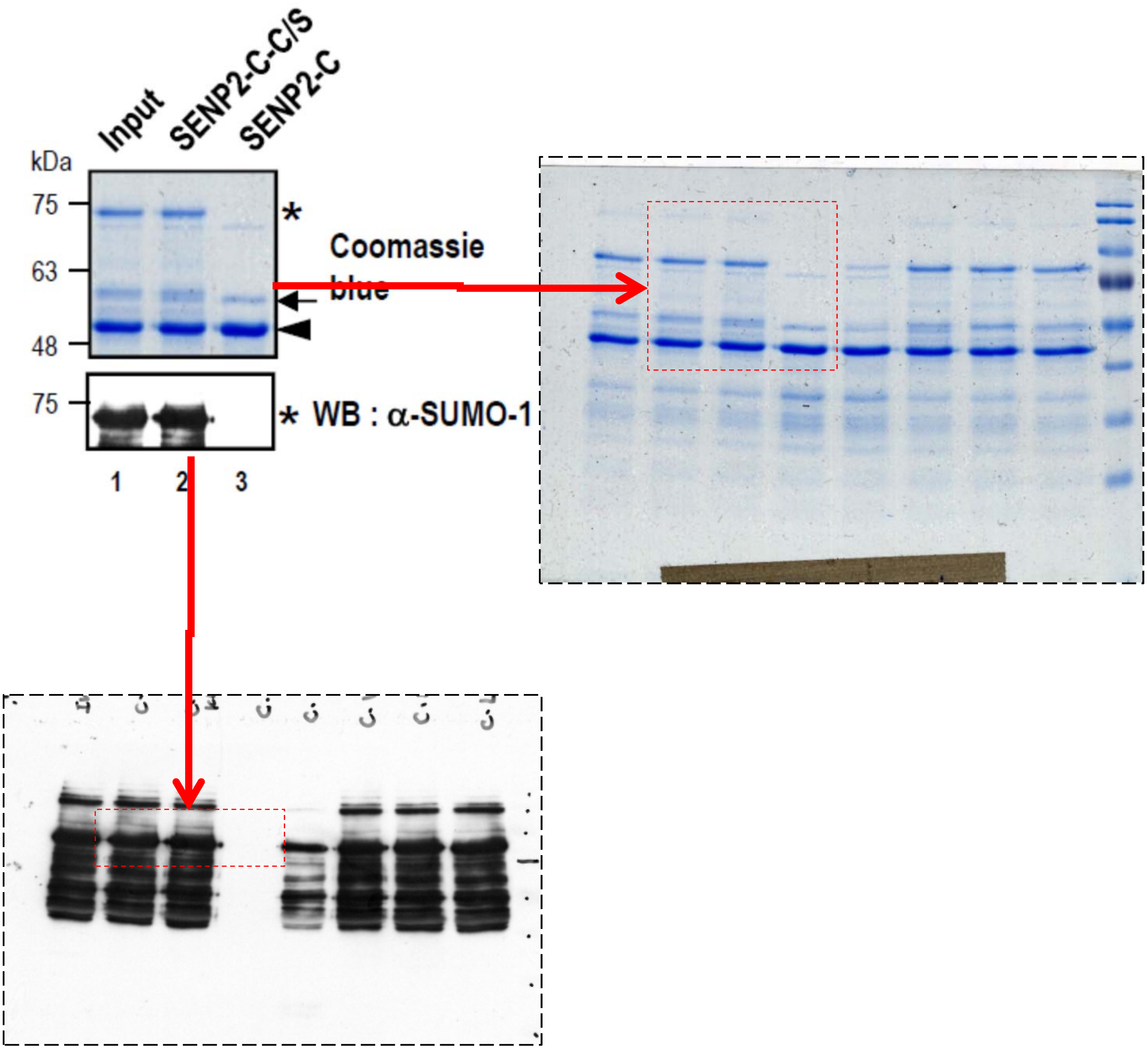

Fig. 2E

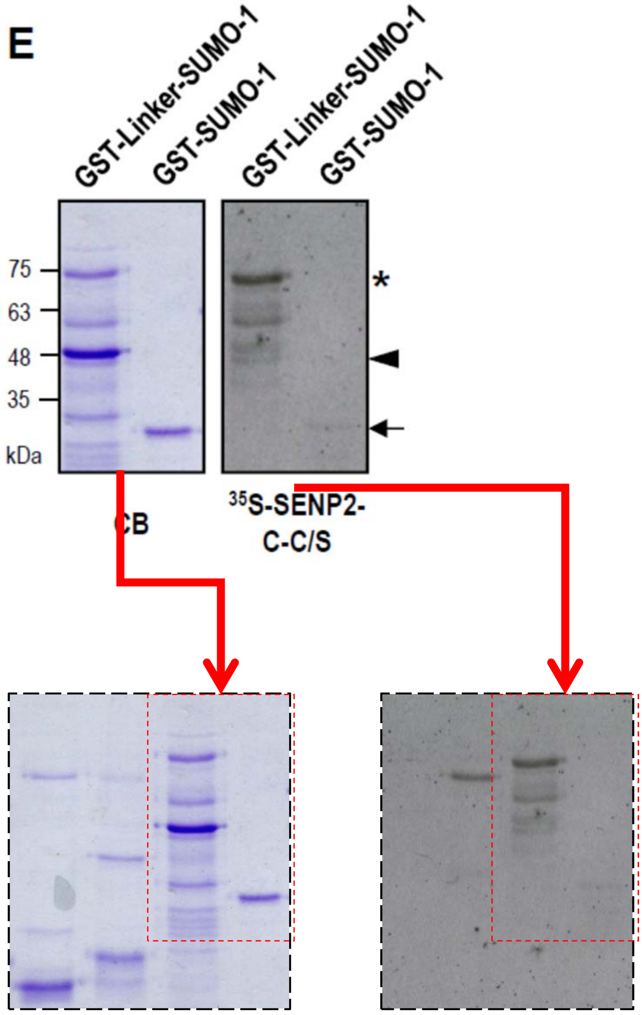

E
GST-Linker-SUMO-1
GST-SUMO-1
GST-Linker-SUMO-1
GST-SUMO-1
75
63
48
35
kDa
*
CB
35S-SENP2-
C-C/S

Fig. 3B

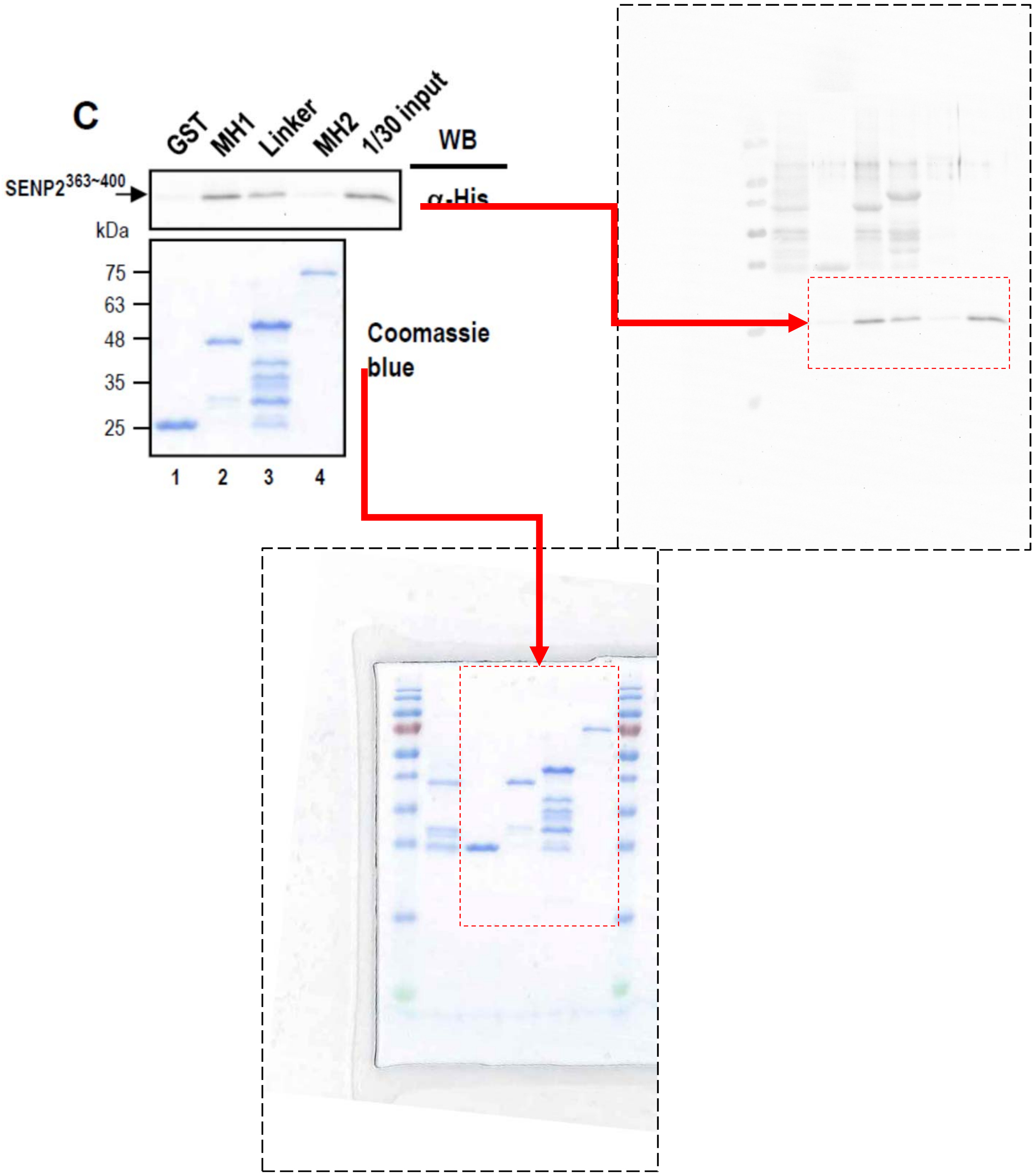
C
GST
MH1
Linker
MH2
1/30 input
WB
SENP2363~400
α-His
kDa
75
63
48
35
25
Coomassie blue
1
2
3
4

Fig. 3C

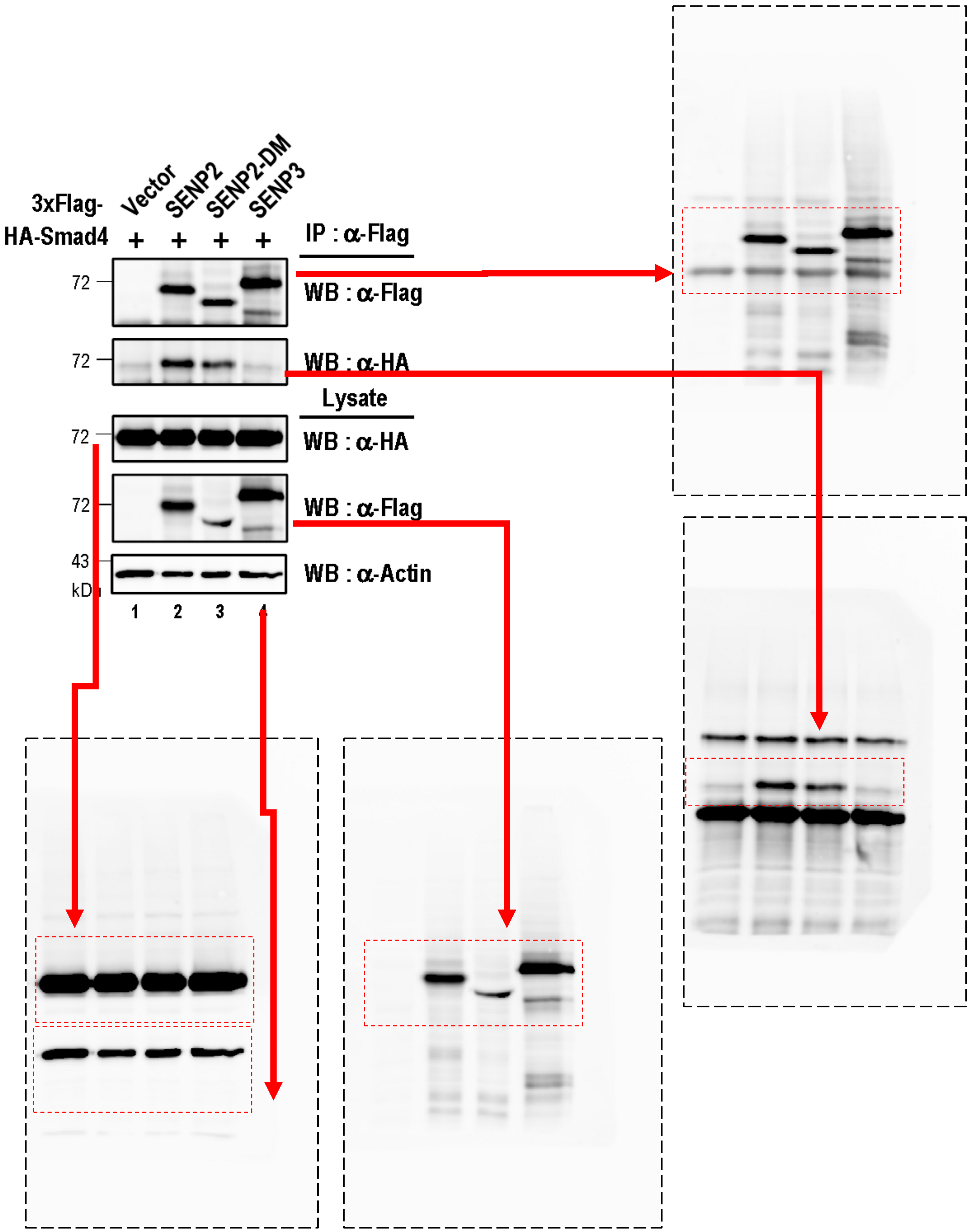
3xFlag-
Vector
SENP2
SENP2-DM
SENP3
HA-Smad4 + + + +
IP : α-Flag
72
WB : α-Flag
72
WB : α-HA
Lysate
72
WB : α-HA
72
WB : α-Flag
43
kDa
WB : α-Actin
1 2 3 4

Fig. 3D

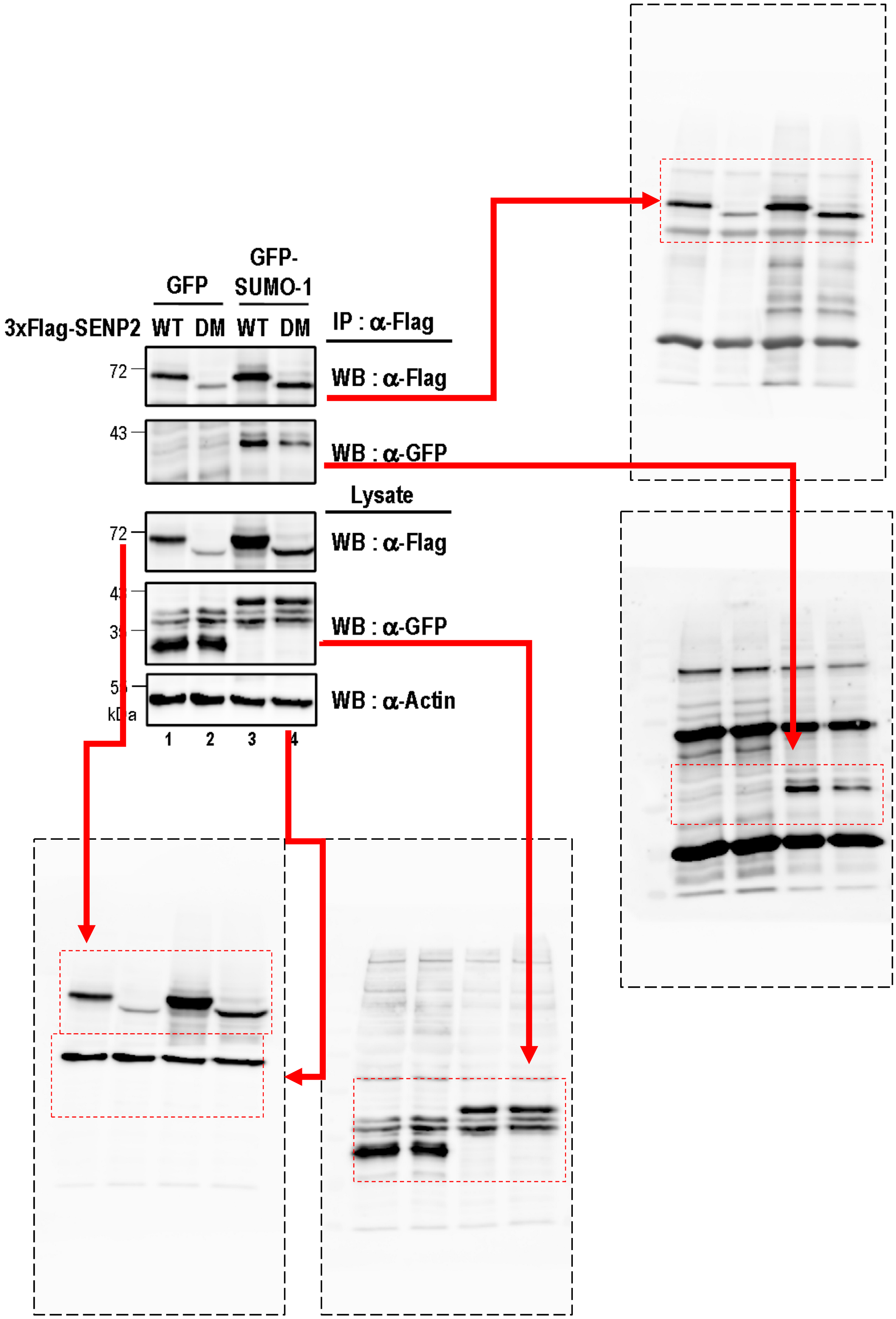
GFP
GFP-SUMO-1
3xFlag-SENP2 WT DM WT DM
IP : α-Flag
72
WB : α-Flag
43
WB : α-GFP
Lysate
72
WB : α-Flag
WB : α-GFP
WB : α-Actin
kDa
1 2 3 4

Fig. 3F

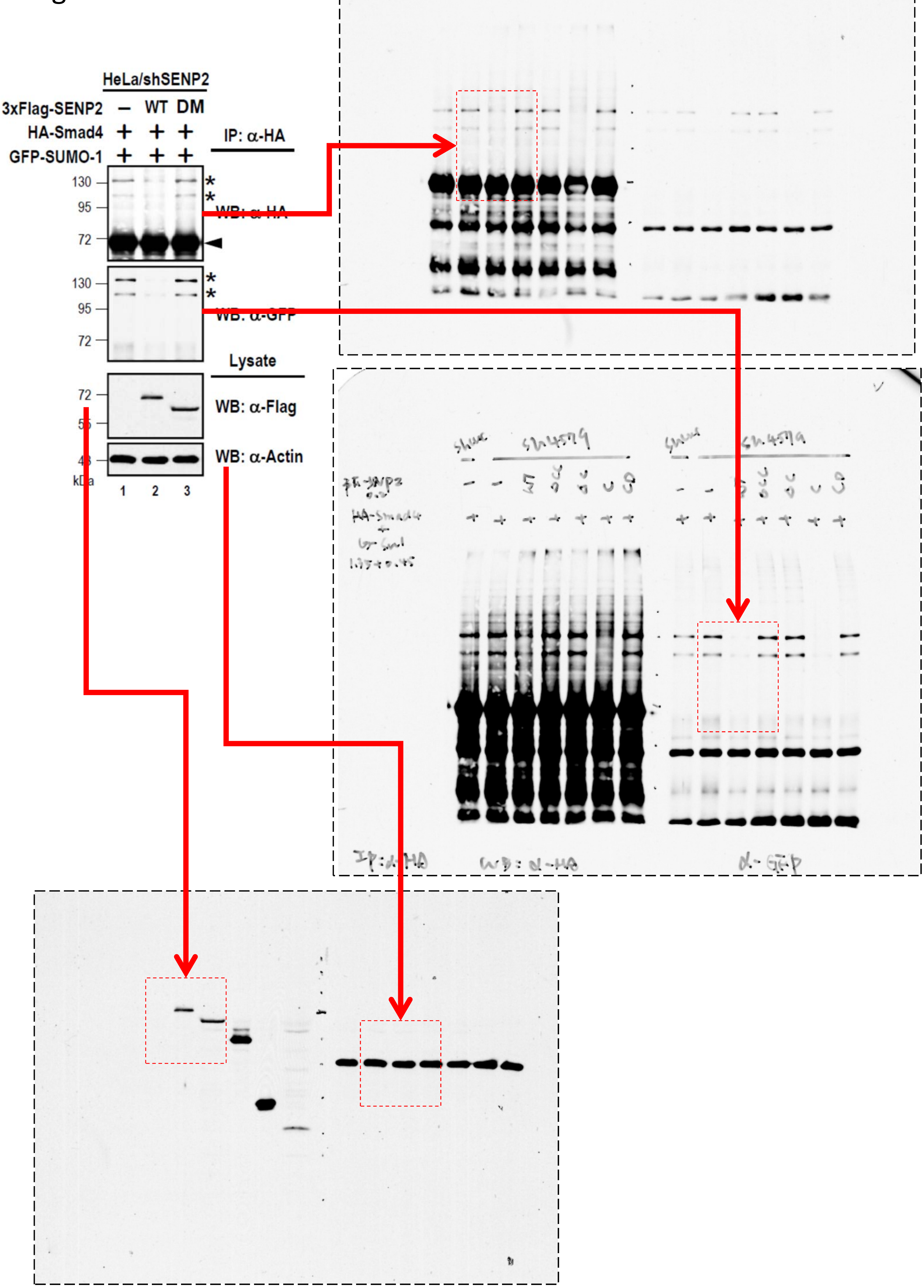

HeLa/shSENP2
3xFlag-SENP2 – WT DM
HA-Smad4 + + +
GFP-SUMO-1 + + +
IP: α-HA
130
95
72
WB: α-HA
130
95
72
WB: α-GFP
Lysate
72
55
WB: α-Flag
43
WB: α-Actin
kDa
1 2 3

# Fig. 3G

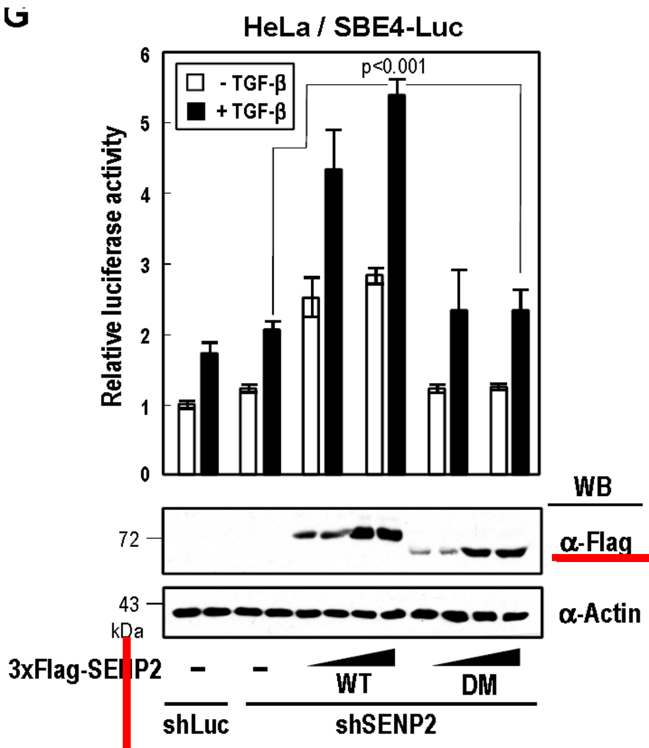
G
HeLa / SBE4-Luc
- TGF-β
+ TGF-β
p<0.001
Relative luciferase activity
6
5
4
3
2
1
0
WB
72
α-Flag
43
kDa
α-Actin
3xFlag-SENP2
WT
DM
shLuc
shSENP2

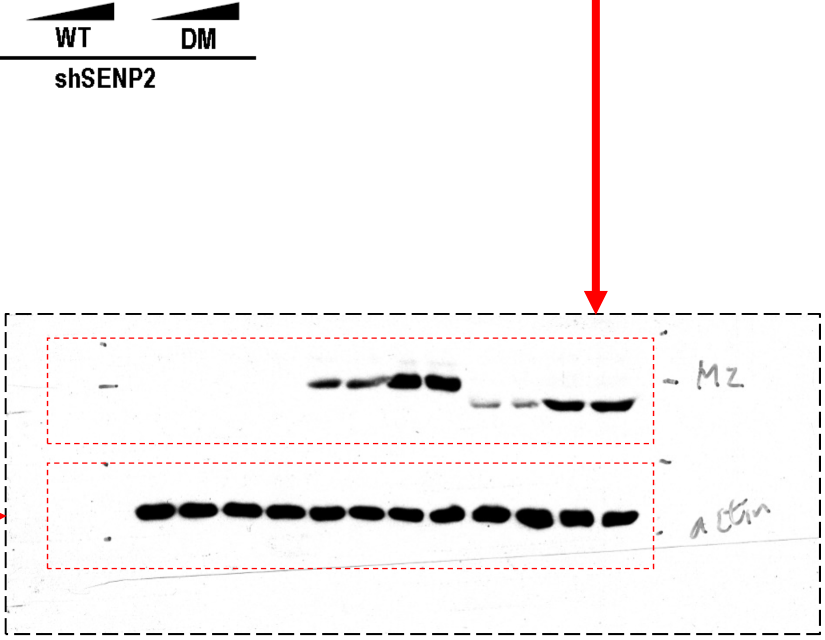

Fig. 4E

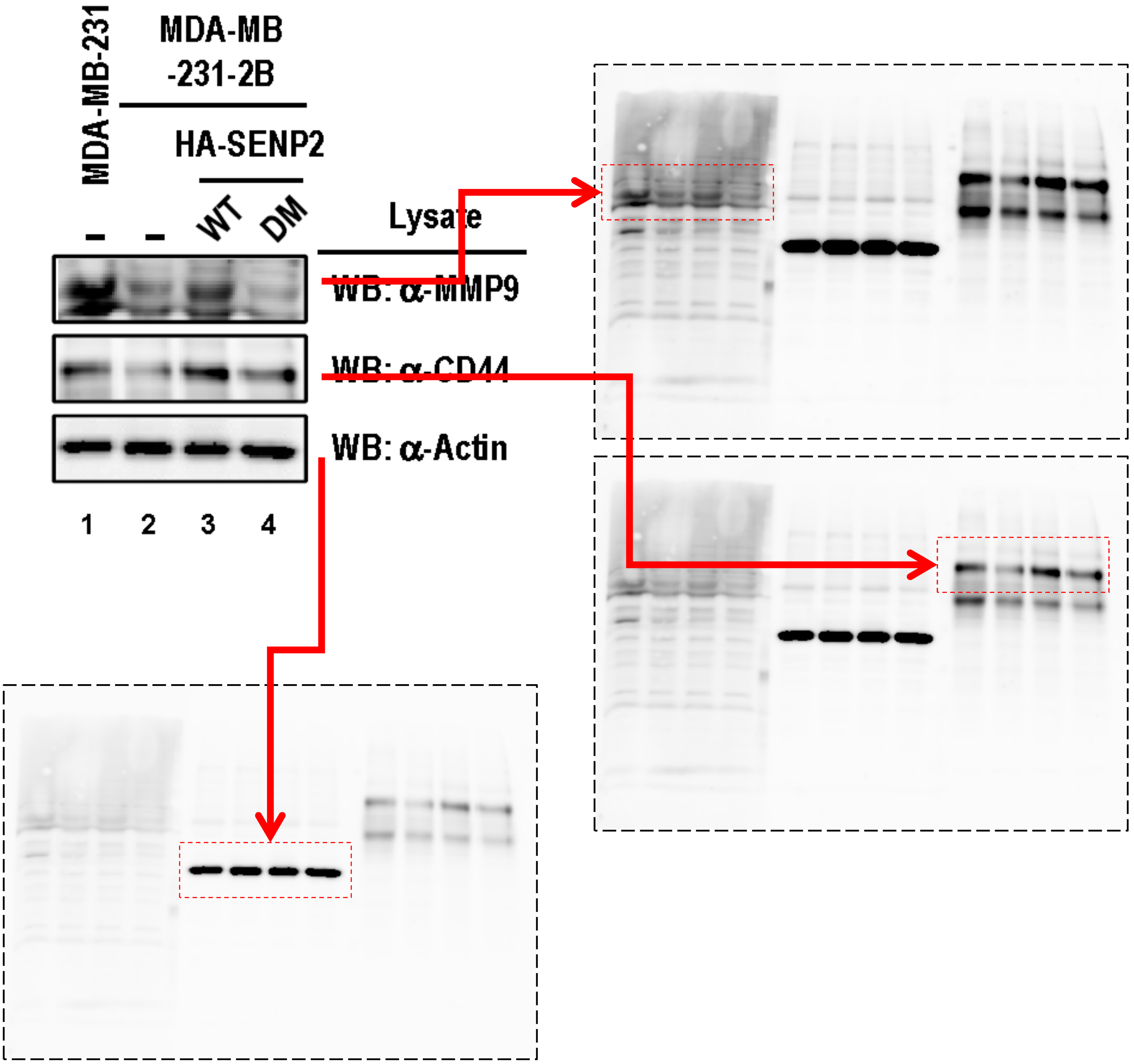
MDA-MB-231
MDA-MB
-231-2B
HA-SENP2
–
–
WT
DM
Lysate
WB: α-MMP9
WB: α-CD44
WB: α-Actin
1
2
3
4

Fig. S1A

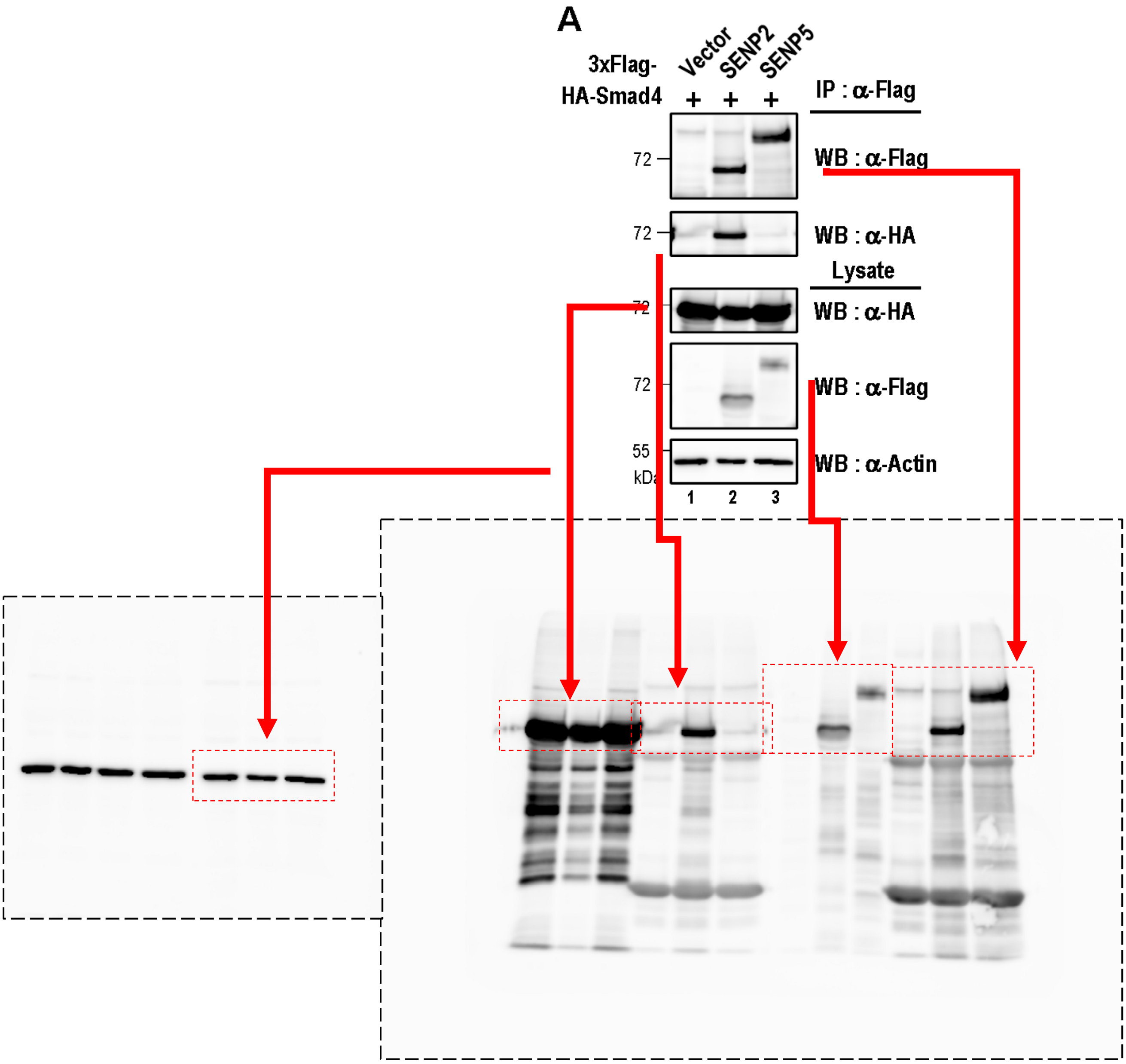
A
3xFlag-
HA-Smad4
Vector
SENP2
SENP5
+ + +
IP : α-Flag
72
WB : α-Flag
72
WB : α-HA
Lysate
72
WB : α-HA
72
WB : α-Flag
55
kDa
WB : α-Actin
1 2 3

Fig. S1C

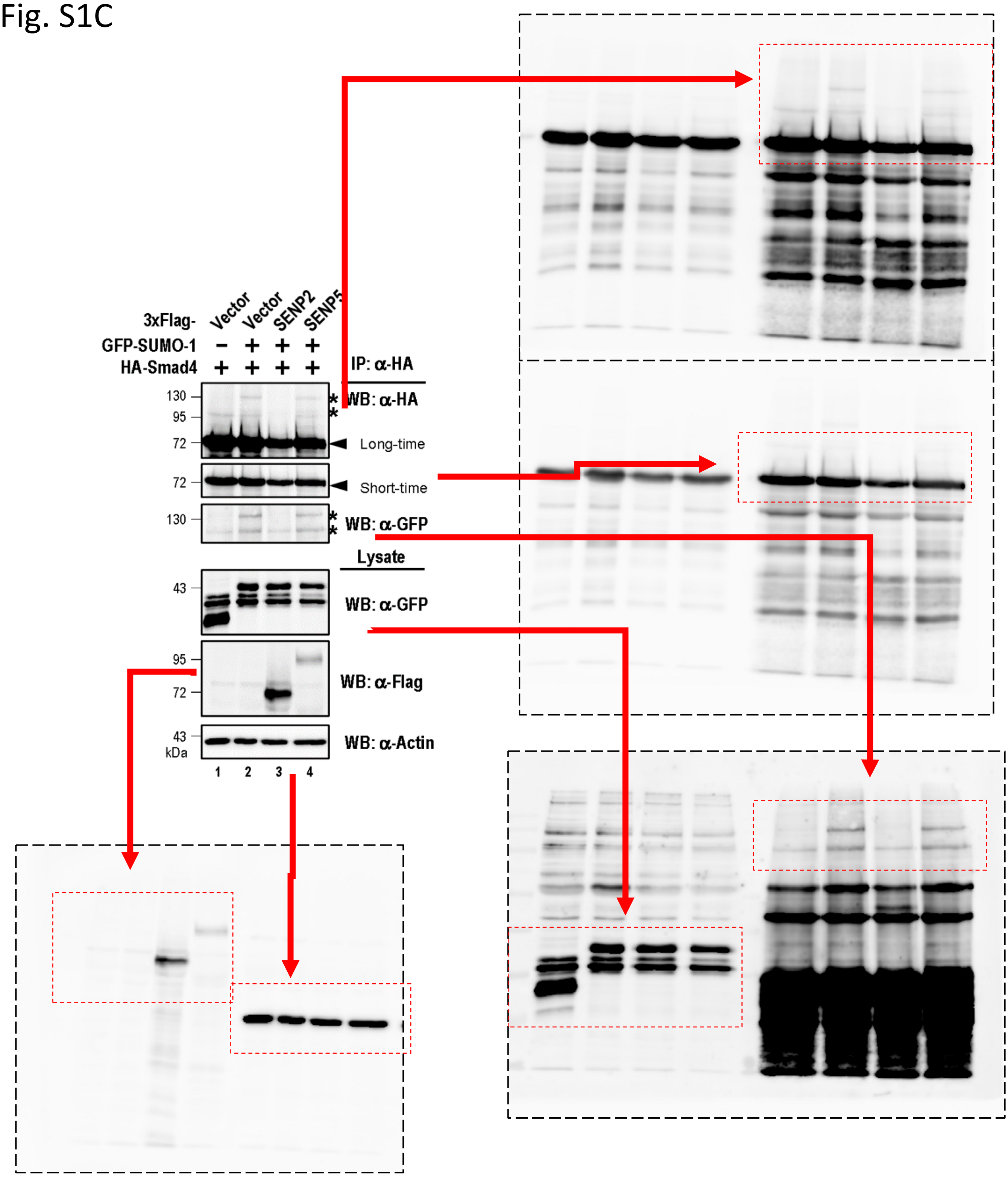

3xFlag-
Vector
Vector
SENP2
SENP5
GFP-SUMO-1
HA-Smad4
IP: α-HA
WB: α-HA
130
95
72
Long-time
72
Short-time
130
WB: α-GFP
Lysate
43
WB: α-GFP
95
72
WB: α-Flag
43
kDa
WB: α-Actin
1
2
3
4
